# Efeitos dos Diferentes Bloqueadores dos Receptores da Angiotensina II Sobre Eventos Cardiovasculares Adversos Maiores: Revisão Sistemática e Metanálise em Rede

**DOI:** 10.36660/abc.20260274

**Published:** 2026-10-21

**Authors:** Eurico Joaquim Lutucuta Ernesto, Ana Claudia Cavalcante Nogueira, Luis Gustavo Sedenho-Prado, Louise Buonalumi Tácito Yugar, Marília Paiva Martins, Daniel Campos Jesus, Jairo Lins Borges, Wilson Nadruz, Luiz Sérgio F Carvalho, Andrei C. Sposito

**Affiliations:** 1 Universidade Estadual de Campinas Campinas SP Brasil Universidade Estadual de Campinas (UNICAMP), Campinas, SP – Brasil; 2 Universidade de Brasília Brasília DF Brasil Universidade de Brasília, Brasília, DF – Brasil; 3 Hospital de Base do Distrito Federal Brasília DF Brasil Hospital de Base do Distrito Federal, Brasília, DF – Brasil; 4 Instituto Aramari Apo Brasília DF Brasil Instituto Aramari Apo, Brasília, DF – Brasil; 5 Universidade Federal de São Paulo São Paulo SP Brasil Universidade Federal de São Paulo (UNIFESP), São Paulo, SP – Brasil; 6 Universidade Católica de Brasília Taguatinga DF Brasil Universidade Católica de Brasília, Taguatinga, DF – Brasil

**Keywords:** Bloqueadores do Receptor Tipo 1 de Angiotensina II, Doenças Cardiovasculares, Metanálise em Rede

## Abstract

**Fundamento:**

Os bloqueadores dos receptores da angiotensina II (BRAs) antagonizam o receptor AT1 e são anti-hipertensivos eficazes. Diferenças modestas na eficácia da redução da pressão arterial têm sido relatadas, refletindo variações na farmacocinética, na ligação ao receptor, na duração da ação e em potenciais efeitos pleiotrópicos. Entretanto, os dados sobre seus efeitos comparativos nos desfechos cardiovasculares em pacientes são escassos.

**Objetivo:**

Avaliar os efeitos dos BRAs sobre eventos cardiovasculares adversos maiores (MACE), hospitalização por insuficiência cardíaca (HHF) e mortalidade por todas as causas em ensaios randomizados não delineados primariamente para avaliar insuficiência cardíaca (IC).

**Métodos:**

As bases de dados MEDLINE/PubMed, Cochrane Central Register of Controlled Trials e ClinicalTrials.gov foram pesquisadas até maio de 2025. Foram incluídos ensaios clínicos randomizados publicados que relataram eventos cardiovasculares adversos maiores (MACE), HHF ou morte por todas as causas, envolvendo BRAs, com pelo menos 100 indivíduos por braço e acompanhamento ≥12 meses. Estudos focados principalmente em pacientes com IC preexistente foram excluídos. Taxas de incidência de HHF, MACE e morte por todas as causas. Foi realizada uma metanálise em rede de efeitos aleatórios, incluindo 27 ensaios clínicos randomizados e 702.584 pacientes-ano.

**Resultados:**

O candesartana reduziu MACE em comparação ao placebo (OR 0,79; IC 95%, 0,63–0,97; SUCRA=0,85), apresentando a melhor classificação entre os BRAs, enquanto a telmisartana demonstrou uma redução menor, porém significativa (OR 0,90; IC 95%, 0,80–0,99; SUCRA=0,61). A análise baseou-se em dados em nível de estudo; as redes de tratamentos incluídas eram moderadamente esparsas, com comparações predominantemente indiretas entre os BRAs.

**Conclusão:**

Esta metanálise em rede, realizada em pacientes sem IC basal, indica que a candesartana e a telmisartana reduzem MACE, sugerindo diferenças clinicamente relevantes entre os BRAs. Considerando a natureza predominantemente indireta de várias comparações, esses achados devem ser interpretados com cautela.

## Graphical abstract

**Figura Central:**
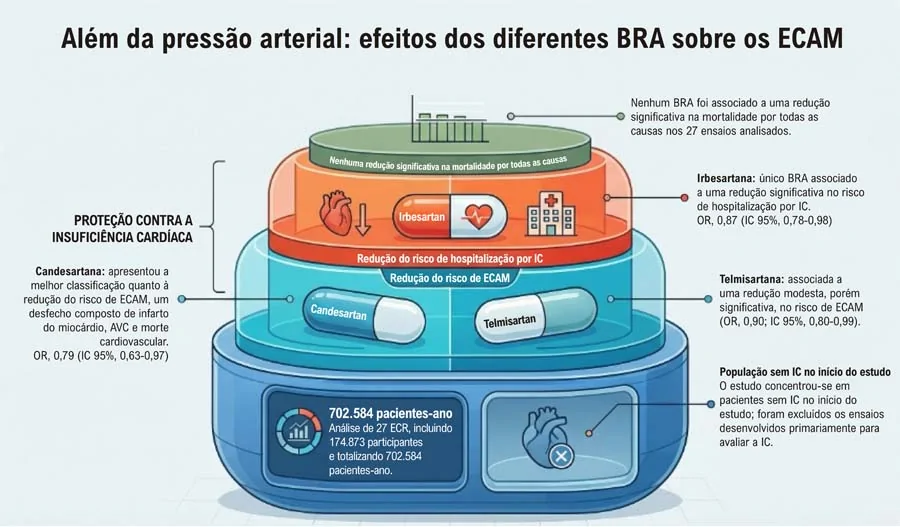
Efeitos dos Diferentes Bloqueadores dos Receptores da Angiotensina II Sobre Eventos Cardiovasculares Adversos Maiores: Revisão Sistemática e Metanálise em Rede

AVC: acidente vascular cerebral; BRA: bloqueadores dos receptores da angiotensina II; ECAM: eventos cardiovasculares adversos maiores; ECR: ensaios clínicos randomizados; IC: insuficiência cardíaca; IC 95%: intervalo de confiança de 95%; OR: odds ratio.

## Introdução

Os bloqueadores dos receptores da angiotensina II (BRA) constituem uma classe farmacológica caracterizada pelo antagonismo seletivo do receptor tipo 1 da angiotensina II (AT_1_R). Embora todos os BRA reduzam eficazmente a pressão arterial (PA), ensaios com comparações diretas e metanálises sugerem diferenças modestas na eficácia anti-hipertensiva e no controle da PA ao longo de 24 horas.^1^ Também foram observadas diferenças clinicamente relevantes em suas propriedades farmacocinéticas, características de ligação ao receptor, duração de ação e possíveis efeitos pleiotrópicos.^1-3^ Por exemplo, a candesartana exerce antagonismo insuperável e apresenta baixa velocidade de dissociação, resultando em bloqueio do receptor que persiste além de sua meia-vida plasmática. Em contrapartida, a losartana atua como antagonista competitivo, enquanto seu metabólito ativo (i.e., EXP 3174), assim como a irbesartana e a valsartana, apresenta perfis antagônicos intermediários. Análises estruturais e simulações de dinâmica molecular demonstraram que, apesar de compartilharem um sítio de ligação comum no AT_1_R, os BRA apresentam interações espaço-temporais e padrões de ligação distintos, que podem explicar as diferenças em suas propriedades farmacodinâmicas e eficácia clínica.^3^ Essas distinções são corroboradas por ensaios vasculares in vitro e estudos de ligação de radioligantes e podem influenciar os desfechos clínicos, incluindo a duração dos efeitos terapêuticos e o controle da PA.^2,4-6^

A inflamação excessiva também contribui para desfechos adversos nas doenças cardiovasculares. Os BRA e os inibidores da enzima conversora de angiotensina (ECA) demonstraram efeitos protetores sobre o endotélio coronariano e o epitélio pulmonar, particularmente em pacientes com condições inflamatórias agudas ou infecções virais. Esses efeitos pleiotrópicos podem contribuir para diferenças na eficácia clínica que vão além do controle da PA. Esta revisão sistemática e metanálise em rede (MAR) teve como objetivo comparar os efeitos dos diferentes BRA sobre os riscos de hospitalização por insuficiência cardíaca (IC), eventos cardiovasculares adversos maiores (ECAM) e mortalidade por todas as causas.

## Métodos

Esta revisão sistemática foi conduzida de acordo com as diretrizes Preferred Reporting Items for Systematic Reviews and Meta-Analyses. O protocolo foi registrado no PROSPERO sob o código CRD42024596628. Como esta metanálise utilizou exclusivamente dados extraídos de estudos publicados anteriormente, não foram necessárias a aprovação por um comitê de ética em pesquisa nem a obtenção de consentimento informado.

### Estratégia de busca e elegibilidade dos estudos

Os detalhes das fontes de dados e da estratégia de busca são apresentados no Material Suplementar. Os critérios de elegibilidade foram estabelecidos de acordo com a estrutura população/intervenção/comparador/desfecho: a população foi constituída por pacientes com hipertensão ou alto risco cardiovascular sem IC no início do estudo; as intervenções foram os diferentes BRA; os comparadores foram placebo ou inibidores da ECA; e os desfechos foram ECAM, hospitalização por IC e mortalidade por todas as causas.

Foram realizadas buscas no MEDLINE via PubMed, ClinicalTrials.gov, Cochrane Central Register of Controlled Trials, Embase, European Union Clinical Trials Register e International Clinical Trials Registry Platform da Organização Mundial da Saúde, desde a criação de cada base até maio de 2025. A inclusão foi restrita a ensaios clínicos randomizados (ECR) publicados na íntegra. A estratégia de busca inicial combinou termos relacionados à hipertensão e à terapia anti-hipertensiva. Para a presente análise, os critérios de elegibilidade foram restringidos a ensaios que avaliaram BRA.

O protocolo original concentrou-se nas comparações entre os BRA. Contudo, a restrição da MAR exclusivamente a ensaios com BRA resultou em duas sub-redes desconectadas, limitando as comparações indiretas entre diversos agentes. Após a inspeção da geometria da rede, foram incorporados ensaios que utilizaram inibidores da ECA como comparadores, a fim de estabelecer uma rede de evidências conectada e possibilitar comparações indiretas entre os BRA, preservando-se o foco principal na terapia com essa classe farmacológica. Essa abordagem fundamentou-se nos pressupostos de transitividade e similaridade entre os ensaios de BRA e de inibidores da ECA quanto às características basais das populações, ao risco cardiovascular e aos objetivos terapêuticos. A inclusão de inibidores da ECA como comparadores também foi considerada biologicamente plausível, uma vez que ambas as classes terapêuticas modulam o sistema renina-angiotensina-aldosterona.

Os estudos foram considerados elegíveis quando i) apresentaram delineamento randomizado, duplo-cego e controlado; ii) avaliaram um BRA ou inibidor da ECA; iii) relataram ECAM, hospitalização por IC ou mortalidade por todas as causas; iv) tiveram seguimento de pelo menos 12 meses; e v) incluíram pelo menos 100 participantes em qualquer grupo randomizado. Foram excluídos os ensaios desenvolvidos primariamente para avaliar a IC, a fim de reduzir a heterogeneidade clínica. As definições dos desfechos são apresentadas na Tabela S4.

### Extração dos dados e avaliação da qualidade

Os detalhes dos procedimentos de extração dos dados e avaliação da qualidade são apresentados no Material Suplementar. Os dados foram extraídos de forma independente por cinco revisores (EJLE, LGSP, LBTY, MPM e DCJ) e posteriormente revisados por ACN. As divergências foram resolvidas por meio de discussão. Os dados extraídos incluíram as características dos estudos, as características basais dos participantes e os desfechos clínicos.

A qualidade dos estudos e o risco de viés foram avaliados por meio da ferramenta Cochrane Risk of Bias 2 (RoB 2). A certeza das evidências para cada desfecho foi avaliada por meio da abordagem Grading of Recommendations Assessment, Development and Evaluation (GRADE), adaptada para MAR. As evidências foram avaliadas em cinco domínios: risco de viés, inconsistência, evidência indireta, imprecisão e viés de publicação. A certeza das evidências foi classificada como alta, moderada, baixa ou muito baixa.

### Síntese dos dados e análise estatística

O desfecho primário foi a ocorrência de ECAM, definidos como um desfecho composto de infarto do miocárdio, acidente vascular cerebral (AVC) e morte cardiovascular. Os desfechos secundários foram hospitalização por IC e mortalidade por todas as causas. Foi realizada uma MAR para estimar as comparações diretas e indiretas entre os tratamentos por meio do pacote “discomb” no R.

As variáveis categóricas são apresentadas como números e porcentagens, enquanto as variáveis contínuas são apresentadas como média ± desvio-padrão (DP) ou mediana (intervalo interquartil [IIQ]), conforme apropriado. As características basais foram calculadas por meio de estimativas ponderadas. Os efeitos dos tratamentos foram expressos como *odds ratios* (OR) na MAR de efeitos aleatórios e como razões de risco nas metanálises pareadas de efeitos aleatórios.

A superfície sob a curva de classificação cumulativa (SUCRA) foi calculada para cada tratamento e desfecho. As classificações relativas dos tratamentos foram sintetizadas por meio dos valores da SUCRA, que refletiram a probabilidade de cada intervenção estar entre os tratamentos mais eficazes. Foram elaborados rankogramas para representar a distribuição das probabilidades de classificação dos tratamentos para cada desfecho. Os efeitos das comparações pareadas entre os tratamentos também foram sintetizados em tabelas comparativas, que apresentaram as OR e os respectivos intervalos de confiança de 95% (IC 95%) para todas as comparações disponíveis na MAR.

A heterogeneidade estatística entre os ensaios foi avaliada por meio da estatística I^2^ de Higgins, com IC 95%. A estatística I^2^ foi derivada do Q de Cochran pela fórmula 100 × (Q − gl)/Q e representou a proporção da variação total atribuível à heterogeneidade entre os ensaios. Em razão da geometria moderadamente esparsa da rede e do número limitado de ciclos fechados em diversas comparações entre tratamentos, a inconsistência foi avaliada qualitativamente por meio da inspeção da estrutura da rede, da comparação entre as estimativas diretas e indiretas, quando possível, e da avaliação da estabilidade dos resultados nas análises de sensibilidade. Não foram realizados testes estatísticos formais de inconsistência.

As possíveis fontes de heterogeneidade entre os ECR foram investigadas por meio de análises de metarregressão. As covariáveis pré-especificadas incluíram tamanho da amostra, duração da intervenção, idade, sexo, raça e etnia, índice de massa corporal (IMC), diagnóstico e estágio da doença renal crônica (DRC), eventos cardiovasculares prévios e PA sistólica (PAS) basal. O viés de publicação foi avaliado por meio de gráficos de funil e do teste de Egger.

A robustez dos achados foi avaliada repetindo-se a MAR sob pressupostos alternativos. Inicialmente, foram excluídos os ECR que compararam inibidores da ECA com placebo. Em seguida, foi realizada uma análise de sensibilidade com exclusão aleatória de 25% dos ensaios para cada desfecho. Nessa análise, foram excluídos aleatoriamente seis ensaios que relataram mortalidade por todas as causas, seis que relataram ECAM e cinco que relataram hospitalização por IC, após o que as OR combinadas e os respectivos IC 95% foram recalculados. Também foram realizadas análises de metarregressão para investigar se as diferenças na PA alcançada entre os grupos de tratamento ativo e controle estavam associadas à incidência dos desfechos clínicos para cada fármaco.

Todos os testes estatísticos foram bicaudais, e valores de p < 0,05 foram considerados estatisticamente significativos. A extração dos dados e as avaliações da qualidade foram submetidas a uma revisão adicional, e as divergências remanescentes foram resolvidas por meio de discussão com pesquisadores seniores. As análises foram realizadas no R, versão 4.0.1 (R Foundation for Statistical Computing), com os pacotes “discomb” (versão 1.0.0), “metaviz” (versão 0.3.1) e “metafor” (versão 2.4.0).

## Resultados

As buscas nas bases de dados identificaram 3.642 registros. Após a remoção de 347 duplicatas, restaram 3.295 registros para triagem. Destes, 2.242 foram excluídos após a triagem dos títulos e resumos, restando 1.053 relatos para avaliação do texto completo. Após a análise dos textos completos, foram incluídos 27 ECR (Figura S1). O número de ensaios que contribuíram para cada rede variou de acordo com o relato dos desfechos: 27 contribuíram para a análise de ECAM, 19 para a análise de hospitalização por IC e 27 para a análise de mortalidade por todas as causas.

As características basais dos participantes dos ECR incluídos são apresentadas na Tabela S1. As definições e os componentes dos ECAM nos ensaios incluídos, bem como sua designação como desfecho primário ou secundário em cada estudo, são sintetizados na Tabela S4. A maioria dos estudos foi classificada como apresentando baixo risco de viés segundo a ferramenta RoB 2 (Tabela S2), e a maioria das comparações específicas por desfecho foi classificada como apresentando evidências de alta certeza segundo a abordagem GRADE (Tabela S5). Os testes de Egger não indicaram viés de publicação significativo para hospitalização por IC (p = 0,16), ECAM (p = 0,22) ou mortalidade por todas as causas (p = 0,16) (Tabela S3 e Figuras S3A-S3C).

As redes de evidências para ECAM, hospitalização por IC e mortalidade por todas as causas concentraram-se predominantemente em comparações controladas por placebo, com relativamente poucas comparações diretas entre os BRA (Figura S2). Consequentemente, a maioria das estimativas comparativas foi obtida indiretamente por meio do placebo ou, em menor grau, de inibidores da ECA como comparadores. Embora placebo, losartana, ramipril, telmisartana e valsartana tenham constituído os nós de tratamento com maior número de conexões, as evidências relativas a diversos BRA foram provenientes de um número limitado de estudos. Isso resultou em uma geometria de rede moderadamente esparsa e em maior dependência de evidências indiretas para algumas comparações pareadas. A avaliação qualitativa da geometria da rede e da coerência das estimativas de efeito não revelou inconsistências importantes nas redes analisadas. A lista completa dos ECR incluídos e suas respectivas referências é apresentada na Tabela S1.

A metanálise incluiu 174.873 participantes, com seguimento médio de 3,1 ± 1,4 anos para o desfecho primário, totalizando 702.584 pacientes-ano. A média de idade dos participantes foi de 65 ± 4 anos; 33% eram mulheres e 67% eram brancos. Os participantes apresentavam diagnóstico de hipertensão havia, em média, 9 ± 4 anos. Além disso, 35% apresentavam diabetes melito tipo 2 e 46%, dislipidemia. No total, 48% dos participantes foram incluídos em ensaios de prevenção secundária, principalmente em razão de doença arterial coronariana ou AVC prévio, enquanto 52% foram incluídos em ensaios de prevenção primária.

No início dos estudos, o IMC médio entre os ensaios foi de 28,5 ± 4,1 kg/m^2^, a PAS média foi de 141 ± 14 mmHg, a PA diastólica média foi de 82 ± 7 mmHg, a fração de ejeção do ventrículo esquerdo (FEVE) média foi de 56% ± 9% e a taxa de filtração glomerular estimada média foi de 73 ± 11 mL/min/1,73 m^2^.

Além dos BRA, estavam disponíveis dados de comparadores provenientes de ECR que avaliaram anlodipino, atenolol, clortalidona e inibidores da ECA. Como a estratégia de busca não foi desenvolvida para identificar de forma abrangente os ensaios desses agentes, as evidências disponíveis foram insuficientes para avaliar seus efeitos individuais. Portanto, as análises que incluíram esses comparadores foram consideradas exploratórias. Para fins de transparência, os respectivos gráficos de floresta são apresentados na Figura S5.

### Desfecho primário: eventos cardiovasculares adversos maiores

A análise incluiu 27 ensaios e demonstrou heterogeneidade moderada (I^2^ = 41%). Em comparação com placebo, a candesartana foi associada a uma redução significativa no risco de ECAM (OR, 0,79; IC 95%, 0,63-0,97; p = 0,03; SUCRA = 0,85). A telmisartana também foi associada a uma redução menor, porém significativa, no risco de ECAM (OR, 0,90; IC 95%, 0,80-0,99; p = 0,02; SUCRA = 0,61). Nenhum outro agente foi associado a uma redução significativa no risco de ECAM (Figura 1A).

**Figura 1:**
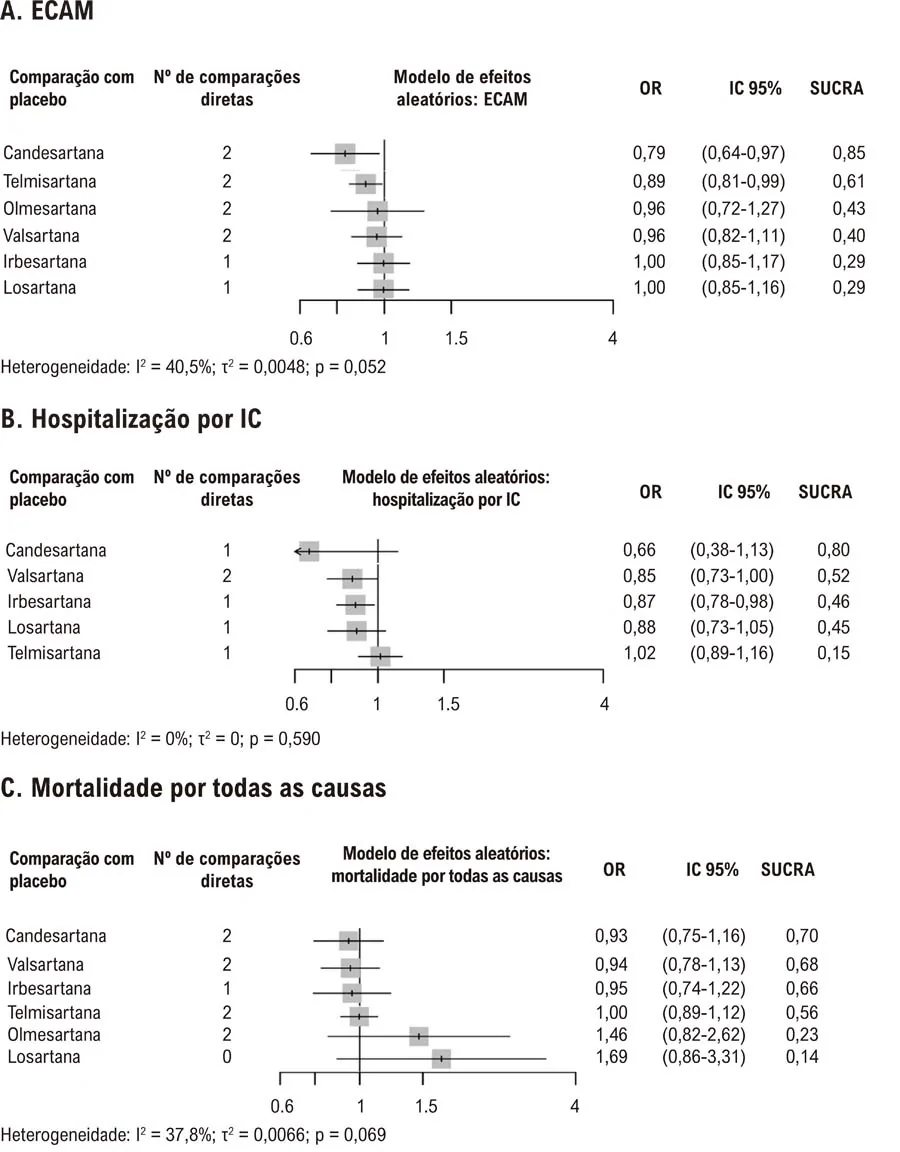
– Forest plots de ECAM, hospitalização por IC e mortalidade por todas as causas. ECAM: eventos cardiovasculares adversos maiores; IC: insuficiência cardíaca; IC 95%: intervalo de confiança de 95%; OR: odds ratio.

### Desfechos secundários

A MAR da hospitalização por IC incluiu 19 ensaios e não demonstrou heterogeneidade estatística (I^2^ = 0%). A avaliação qualitativa não revelou inconsistências importantes entre as evidências diretas e indiretas. Embora a irbesartana tenha apresentado apenas probabilidade moderada de benefício segundo sua classificação entre os tratamentos (SUCRA = 0,46), foi o único agente associado a uma redução significativa no risco de hospitalização por IC (OR, 0,87; IC 95%, 0,78-0,98; p = 0,02). Esse achado deve ser interpretado com cautela, pois foi determinado predominantemente por um único ensaio e respaldado por evidências de certeza moderada segundo a avaliação GRADE.

A análise da mortalidade por todas as causas incluiu 27 ensaios e demonstrou heterogeneidade baixa a moderada (I^2^ = 38%). Nenhum tratamento foi associado a diferença significativa na mortalidade por todas as causas em comparação com placebo (Figura 1C).

Os rankogramas de ECAM e mortalidade por todas as causas são apresentados na Figura S9, e todas as comparações pareadas são apresentadas nas Tabelas S6 e S7.

### Análises de sensibilidade e de metarregressão

Em razão da heterogeneidade moderada observada para ECAM e da diversidade das populações, dos comparadores e dos delineamentos dos estudos incluídos, foram realizadas diversas análises de sensibilidade para avaliar a robustez dos principais achados.

Conforme demonstrado na Figura S4, a análise de sensibilidade com exclusão aleatória de 25% dos ensaios produziu resultados consistentes com os da análise principal para todos os desfechos. Da mesma forma, a restrição da análise aos ECR com baixo risco de viés segundo a ferramenta RoB 2 não alterou substancialmente os resultados.

Em outra análise de sensibilidade, foram excluídos os ensaios que compararam inibidores da ECA com placebo (Figura S6). Após a exclusão desses ensaios, a candesartana permaneceu como o único agente significativamente associado a menor risco de ECAM em comparação com placebo (OR, 0,79; IC 95%, 0,66-0,96). Para hospitalização por IC e mortalidade por todas as causas, a exclusão desses ensaios reduziu o poder estatístico, e nenhum agente apresentou associação significativa com qualquer um desses desfechos.

A utilização de um modelo de efeito fixo em vez de um modelo de efeitos aleatórios produziu estimativas consistentes com as da análise principal, inclusive para hospitalização por IC, desfecho para o qual não foi observada heterogeneidade estatística (I^2^ = 0%).

Foram realizadas análises de metarregressão para investigar se a magnitude da redução da PAS alcançada nos grupos de tratamento ativo em relação aos grupos controle influenciou os desfechos clínicos. A inspeção visual dos gráficos de metarregressão para mortalidade por todas as causas e ECAM sugeriu que os efeitos do tratamento da maioria dos BRA, incluindo telmisartana, valsartana e irbesartana, permaneceram relativamente estáveis em toda a amplitude observada das diferenças de PAS entre os grupos (Figura S7). Embora a candesartana e a olmesartana tenham apresentado inclinações mais acentuadas nessas análises exploratórias, os amplos IC indicaram incerteza substancial, e as associações observadas, em geral, não foram estatisticamente significativas.

Além disso, conforme demonstrado na Figura S8, os gráficos de floresta estratificados segundo a magnitude da alteração da PAS (por exemplo, diferenças de PAS entre os grupos ativo e controle [ativo − controle] > −1,3 mmHg, entre −1,3 e −2,9 mmHg e < −2,9 mmHg) demonstraram que os riscos de mortalidade por todas as causas e ECAM permaneceram, em grande parte, consistentes, independentemente da diferença de PA alcançada. O efeito combinado global sobre os ECAM alcançou significância estatística apenas no subgrupo com a maior redução da PAS (*hazard ratio* [HR], 0,92; IC 95%, 0,87-0,97), sugerindo que a redução da PA pode ter contribuído parcialmente para os efeitos observados dos tratamentos.

Adicionalmente, análises abrangentes de metarregressão demonstraram que outros fatores pré-especificados (por exemplo, tamanho da amostra, duração da intervenção, idade, sexo, raça e etnia, IMC, diagnóstico e estágio da doença renal crônica e eventos cardiovasculares prévios) não influenciaram significativamente os resultados dos desfechos primário ou secundários (Tabela S8). Esses achados corroboram a robustez dos benefícios observados com a candesartana e a telmisartana, sugerindo que esses benefícios podem ser atribuídos a propriedades farmacológicas intrínsecas, e não às características basais dos participantes ou apenas ao grau de controle da PA.

## Discussão

Esta MAR fornece uma avaliação quantitativa das associações entre os diferentes BRA e os ECAM em ECR que não foram especificamente desenvolvidos para avaliar a IC. A candesartana e a telmisartana foram associadas a reduções significativas no risco de ECAM, enquanto a irbesartana foi associada a uma redução no risco de hospitalização por IC em análises exploratórias. Contudo, o achado referente à irbesartana deve ser interpretado com cautela, pois foi determinado predominantemente por um único ensaio que incluiu pacientes com diabetes e proteinúria acentuada, o que limita sua generalização. Além disso, a certeza das evidências para esse desfecho foi rebaixada para moderada na avaliação GRADE. Nenhum agente demonstrou benefício significativo sobre a mortalidade. Esses achados levantam a hipótese de que diferenças farmacodinâmicas ou farmacocinéticas entre os BRA possam estar associadas a diferenças nos desfechos clínicos.

As metanálises anteriores concentraram-se principalmente na eficácia dos diferentes BRA na redução da PA ou avaliaram os BRA como uma classe quanto aos desfechos cardiovasculares e renais.^7-10^ Embora alguns estudos tenham quantificado diferenças na redução da PA, como o controle ambulatorial menos eficaz da PA observado com a losartana,^7^ nenhum comparou diretamente os desfechos cardiovasculares entre os diferentes BRA. Até onde sabemos, esta é a primeira MAR a avaliar diferenças na ocorrência de ECAM entre os diferentes BRA.

Conforme observado anteriormente, foram descritas interações espaço-temporais e padrões de ligação distintos entre os BRA.^3^ Essas diferenças podem influenciar tanto a duração dos efeitos terapêuticos quanto o controle da PA.^2,4-6^ Embora todos os BRA bloqueiem seletivamente o AT_1_R, sua afinidade por esse receptor varia substancialmente. Por exemplo, a candesartana apresenta seletividade pelo AT_1_R mais de 10.000 vezes superior à observada pelo receptor tipo 2 da angiotensina II.^6^ Atua como antagonista insuperável, produzindo bloqueio prolongado e não competitivo da vasoconstrição induzida pela angiotensina II.^5,6^ Em contrapartida, a losartana atua como antagonista competitivo, produzindo um deslocamento paralelo da curva de dose-resposta da angiotensina II para a direita, sem reduzir a resposta máxima.^6^ A irbesartana e a valsartana apresentam propriedades intermediárias entre o antagonismo competitivo e o insuperável.^5,6^ Ainda não está claro se essas distinções farmacológicas ou outros mecanismos ainda não identificados são responsáveis pelas diferenças observadas neste estudo. Ainda assim, os presentes achados sugerem que as diferenças farmacológicas entre os BRA podem se traduzir em diferenças clinicamente relevantes nos desfechos.

Este estudo apresenta diversos pontos fortes, incluindo a utilização de uma estrutura de MAR, que possibilitou comparações abrangentes entre os tratamentos, e seu foco específico em pacientes sem IC no início dos estudos. Entretanto, algumas limitações importantes devem ser reconhecidas. Primeiro, a análise baseou-se em dados agregados dos estudos, e não em dados individuais dos participantes, o que impossibilitou a realização de análises detalhadas de subgrupos. Segundo, embora a heterogeneidade estatística tenha sido moderada para ECAM, permaneceu considerável variabilidade clínica e metodológica entre os ensaios incluídos. Apesar da consistência dos resultados em diversas análises de sensibilidade, não se pode excluir a presença de heterogeneidade residual. Terceiro, as redes de tratamentos basearam-se predominantemente em comparações controladas por placebo, com relativamente poucas comparações diretas entre os BRA, resultando em uma geometria de rede moderadamente esparsa. Além disso, foram incorporados ensaios que utilizaram inibidores da ECA como comparadores para preservar a conectividade da rede. Essa abordagem pode ter aumentado a dependência de evidências indiretas e exige cautela na interpretação de algumas comparações. Quarto, as variações no delineamento dos estudos, na duração do seguimento e nas definições dos desfechos entre os ECR podem ter influenciado os achados. Quinto, embora tenham sido excluídos os ensaios especificamente desenvolvidos para avaliar a IC, a FEVE basal e a disfunção ventricular esquerda assintomática não foram relatadas de maneira uniforme. Consequentemente, não se podem excluir diferenças residuais na função ventricular entre os estudos, e não foi possível avaliar sistematicamente uma possível modificação do efeito de acordo com a FEVE basal. Sexto, embora as classificações da SUCRA forneçam uma hierarquia probabilística da eficácia dos tratamentos, devem ser interpretadas com cautela. Valores elevados da SUCRA não indicam necessariamente superioridade clinicamente relevante, particularmente quando as magnitudes dos efeitos são modestas e os intervalos de confiança se sobrepõem. Sétimo, as análises exploratórias de metarregressão sugeriram que a magnitude da redução da PA não explicou integralmente as diferenças observadas nos desfechos entre os BRA. Contudo, a redução da PA pode ter atuado como mediadora parcial, e seu papel não pode ser excluído em razão das limitações inerentes às análises baseadas em dados agregados dos estudos. Por fim, assim como em todas as metanálises, os achados nulos podem refletir a ausência de ECR adequadamente delineados, e não necessariamente a inexistência de um verdadeiro efeito do tratamento.

## Conclusão

Esta MAR sugere a existência de diferenças clinicamente relevantes entre os BRA em pacientes sem IC no início dos estudos, com a candesartana e, em menor grau, a telmisartana associadas a reduções no risco de ECAM. Em razão da natureza predominantemente indireta de várias comparações e da geometria moderadamente esparsa da rede, esses achados devem ser interpretados com cautela e considerados geradores de hipóteses, em vez de evidências definitivas que respaldem a escolha preferencial de um BRA específico. Ainda assim, as diferenças observadas nos desfechos entre os BRA são compatíveis com a diversidade farmacológica dessa classe de medicamentos reconhecida nas diretrizes atuais.

## Supplementary Material

*Material suplementar Para informação adicional, por favor, clique aqui.http://abccardiol.org/supplementary-material/2026/12309/2026-0274-supplement-data.pdf
