# Supplementary material for: Efeitos dos Diferentes Bloqueadores dos Receptores da Angiotensina II Sobre Eventos Cardiovasculares Adversos Maiores: Revisão Sistemática e Metanálise em Rede
Source: Arq Bras Cardiol. 2026 Oct 21;123(9):e20260274. [Article in Portuguese] doi: 10.36660/abc.20260274 (PMC13754392; doi:10.36660/abc.20260274)
Supplement: Supplementary file 1 [file 0066-782x-abc-123-9-e20260274-suppl01-en.pdf]

Angiotensin II Receptor Blockers and their Individual Impact on Cardiovascular Events: A Systematic Review and Network Meta-Analysis

SUPPLEMENTARY DATA

Data Source and Search

Queries of literature were performed using the electronic databases Medline (PubMed), ClinicalTrials.gov, Cochrane Central Register of Controlled Trials, Embase, European Union Clinical Trials Register, and World Health Organization (WHO) International Clinical Trials Registry Platform. The search included all submitted articles until May 1st, 2025, with no restriction to submission date or language. The search was filtered to include only randomized controlled trials (RCTs) involving humans. Systematic reviews and meta-analyses were also evaluated to identify other relevant RCTs.

The following search terms were used:

Category	Search Term / Component	Description	Rationale for Inclusion in a Systematic Review
Disease/Condition		This section defines the patient population of interest.	To focus the search on studies involving patients with the target cardiovascular conditions.
	#1 """hypertension""[MeSH Terms] OR ""blood pressure""[All Fields]"	This combines a specific Medical Subject Heading (MeSH) for hypertension with a broader keyword search for "blood pressure".	The MeSH term retrieves articles specifically indexed under hypertension, ensuring high relevance. The "All Fields" search for "blood pressure" captures a wider net of studies, including those that may not have been assigned the "hypertension" MeSH term but are still relevant.
	#2 """heart failure""[MeSH Terms] OR (""heart""[All Fields] AND ""failure""[All Fields])"	Similar to the hypertension search, this uses the MeSH term for heart failure and a keyword combination for broader coverage.	This strategy ensures the inclusion of studies on patients with heart failure, a common comorbidity and a key condition of interest in cardiovascular research.
	#3 """cardiovascular diseases""[MeSH Terms] OR (""cardiovascular""[All Fields] AND ""diseases""[All Fields])"	This broadens the search to include a wide range of diseases affecting the heart and blood vessels.	This term is crucial for capturing studies that may investigate the effects of the interventions on a spectrum of cardiovascular conditions beyond just hypertension and heart failure.
Intervention		This section specifies the treatments being investigated.	To isolate studies that evaluate the effectiveness of specific classes of antihypertensive medications.
ACE Inhibitors	#4 """angiotensin-converting enzyme inhibitors""[MeSH Terms] OR ""enalapril""[MeSH Terms] OR ""lisinopril""[MeSH Terms] OR ""ramipril""[MeSH Terms] OR ""captopril""[MeSH Terms] OR ""perindopril""[All Fields]"	This targets the class of Angiotensin-Converting Enzyme (ACE) inhibitors and includes several of the most commonly prescribed drugs in this class.	ACE inhibitors are a first-line treatment for hypertension and heart failure. Including both the class name and specific, widely-used examples (Enalapril, Lisinopril, Ramipril, Captopril, Perindopril) increases the search's sensitivity to find all relevant trials.
ARBs	#5 """angiotensin receptor blockers""[MeSH Terms] OR ""losartan""[MeSH Terms] OR ""valsartan""[MeSH Terms] OR ""candesartan""[MeSH Terms] OR ""irbesartan""[MeSH Terms] OR ""olmesartan""[MeSH Terms]"	This focuses on Angiotensin II Receptor Blockers (ARBs), another major class of antihypertensive drugs, and lists common examples.	ARBs work by a different mechanism than ACE inhibitors but have similar indications. Including this class and its key drugs (Losartan, Valsartan, Candesartan, Irbesartan, Olmesartan) is essential for a comprehensive review comparing different renin-angiotensin system inhibitors.
Outcomes		This section defines the clinical endpoints of interest.	To identify studies that report on the clinically meaningful effects of the interventions.
	#9 """myocardial infarction""[MeSH Terms] OR ""stroke""[MeSH Terms] OR ""mortality""[All Fields] OR ""hospitalization""[All Fields]"	This combines MeSH terms for major adverse cardiovascular events (heart attack and stroke) with keywords for all-cause	These are critical "hard" clinical endpoints in cardiovascular trials. Their inclusion ensures that the review will focus on studies measuring the most important outcomes for patients.

		mortality and hospitalization rates.	
Study Design & Filters		This section refines the search to include the most robust type of evidence and relevant population.	To ensure the methodological quality and applicability of the included studies.
	#10 """randomized controlled trials ""[MeSH Terms] OR ""randomized controlled trial""[All Fields] OR controlled clinical trial[Publication Type]) "	This filter is designed to retrieve studies that are randomized controlled trials (RCTs) or controlled clinical trials (CCTs).	RCTs are considered the gold standard for determining the causal effect of an intervention. This filter prioritizes the highest quality of evidence, which is a hallmark of a systematic review.
	#11 """humans""[MeSH Terms]"	This is a standard filter to exclude animal studies and in-vitro research.	This ensures that the retrieved studies are directly applicable to human health and clinical practice.

Data Extraction and Quality Assessment

Data was extracted by multiple authors. Any inconsistencies were resolved after debate with a senior researcher. Extracted data concerning patient characteristics and study results included: year of publication, authors, journal published, study design, characteristic of patients, sample size, duration of intervention, drug dose, and clinical outcomes.

The primary endpoint was defined as Hospitalization for Heart Failure (HHF). Secondary endpoints were Major Adverse Cardiovascular Events (MACE), All-Cause Death, Cardiovascular Death, non-fatal Myocardial Infarction (MI), and non-fatal Stroke.

The quality of the included RCTs was assessed using the Cochrane Risk of Bias 2 (RoB 2) tool for assessing risk of bias.

Table S1. Baseline characteristics of patients in the included randomized controlled trials

RCT	Publishing year	Therapy	Control	Inclusion criteria	Original Primary Outcome	Sample size	Follow-up time (years)	Age (years)	Woman (%)	BMI (Kg/m^2)	SBP (mmHg)	DBP (mmHg)	DM(%)	Hypertension (%)	current smoker (%)
AASK	2002	Ramipril	Amlodipine	Hypertension and GFR 20-65 mL/m2	Change in GFR	653	4	55±11	39.6		151	23	0	100	
			Metoprolol			441	4	54±11	38.5		150	24	0	100	
ALLHAT	2002	Lisinopril	Amlodipine	Hypertension and high CV risk	Composite of CAD or non-fatal MI	18102	4.9	67±8	47.3	29.8	146	84	35.5	100	21.9
			Clortalidone			15255	4.9	67±8	47	29.7	146	84	35.5	100	21.9
AMADEO	2008	Telmisartan	Losartan	T2DM and proteinuria	Change in urinary protein-creatinine	860	1	61±9	35.7	29.9	143.7	79.9	100	100	15
CONSENSUS II	1992	Enalapril	Placebo	MI	Death to any cause	6090	2.1	66	26		133	80	12	NA	37
DIABHYCAR	2004	Ramipril	Placebo	DM	Composite of MACE and ESRF	4912	3.92	65±8	45.929	29.2	145.8	82.2	100	56.4	15.2
EUROPA	2003	Perindopril	Placebo	Albuminuria	Composite of MACE	12218	4.2	60±9	14.7		137	82	11.8	27	
GISSIAF	2009	Valsartan	Placebo	AF and one (HF, Hypertension, T2DM, stroke, PAD, CAD) or AF with LA dilation	Recurrence of AF	1442	1	68±9	38.5	27.7	138.2	81.5	13.2	85.7	7.9
HOPE	2000	Ramipril	Placebo	High CV risk		9297	4.5	66±7	25.8	28	139	79	38.9	47.6	13.9
IDNT	2001	Irbesartan	Placebo	DM, Hypertension and proteinúria (>900 mg in 24h)	Renal composite	1148	2.6	58±8	29	30.5	160	87	100	100	
			Amlodipine			579	2.6	59±8	37	30.9	160	87	100	100	
IMAGINE	2008	Quinapril	Placebo	Post-CABG	Recurrent individual MACE components	2553	2.95	61±10	13		122	70	9	47	20
Kondo et al., 2003	2003	Candesartan	Placebo	Non significant coronary stenosis	Composite of revasculariz ation, non-fatal MI, CV death	406	2	65±10	23	23	129	76	27	48	25
LIFE	2002	Losartan	Atenolol	Hypertension and ECG signs of LVH	Composite of CV death, stroke, and MI	9193	4.8	67±7	54	28	174.3	97.9	13	100	16
NAVIGATOR	2010	Valsartan	Placebo	Impaired glucose tolerance and one or more CV risk factors or known CAD	Coprimary: incidence of diabetes and composite of MACE	9306	5	64±7	51.3	30.6	139.4	82.5	0	77.3	11.2
OLIVUS-Ex	2012	Olmesartan	Placebo	Hypertension and clinical stable angina	Incidence of MACCE	247	3.41	68±9	32	23.9	1424	792	31	100	34
ONTARGET	2008	Ramipril	Telmisartan	Coronary, peripheral, or cerebrovascular disease or diabetes with	Effectivene ss	17118	4.6	66±7	26.3	28.1	141.8	82.1	36.7	69	12.4

				end-organ damage											
OPTIMAAL	2002	Losartan	Captopril	MI	Death to any cause	5477	2.7	67±10	29.3	26.6	123	71.5	17.8	35.8	
PART-2	2000	Ramipril	Placebo	History of PAD, MI, CCS or TIA	Carotid thickness	617	4.7	61±8	18		133	79	8	NA	38
PREAMI	2006	Perindopril	Placebo	MI	Composite of MACE	1252	1	73±5	35	26	126	74	25	59	32
PROGRESS	2001	Perindopril	Placebo	History of stroke or TIA	Fatal or non-fatal stroke	6105	3.9	64±10	30		147	86	13	48	20
PRoFESS	2008	Telmisartan	Placebo	Ischemic stroke (<90 days)	Stroke of any type	20332	2.5	66±9	36.2	26.8	144.1	83.8	28	74	21.2
QUIET	2001	Quinapril	Placebo	Post PCI	Cardiac event	1750	2.25	58	19	NA	123	74	14	48	23
ROADMAP	2011	Olmesartan	Placebo	T2D	Microalbu minuria	4447	4	58±9	54.7	30.9	137	81	100		18.5
SCAT	2000	Enalapril	Placebo	CAD and LVEF >35%	Quantitativ e coronary angiograph y	460	3.98	62±9	89		128	77	11	39	15
SCOPE	2003	Candesartan	Placebo	Hypertension	Composite of MACE	4937	3.7	76	64.2	26.9	166	90.3	12.5	100	8.7
TRANSCEND	2008	Telmisartan	Placebo	Coronary, peripheral, or cerebrovascular disease or diabetes with end-organ damage	Composite of MACE	5926	4.6	67±7	42.6	28.1	140,7	81,8	35.8	76.5	9.9
VALUE	2004	Valsartan	Amlodipine	Hypertension and high CV risk	Composite of MACE	15245	4.2	67±8	42.5	38.6	154.5	87.4	NA	100	
VIVALDI	2008	Telmisartan	Valsartan	Hypertension, DM and proteinuria	Change 24h proteinuria	867	1	65±9	35.8	30.4	147.6	81.6	100	100	18.1

AF: atrial fibrilation; **AMI**: acute myocadial infarction; **ASCVD**: atherosclerotic cardiovascular disease; **CCS**: chronic coronary syndorem; **CABG**: coronary artery bypass graft; **CAD**: coronary arterial disease; **CKD**: chronic kideny disease; **CV**: Cardiovascular; **ESRF:** end stage renal failure; **GFR:** glomerular filtration rate **HF**: heart failure; **LA**: left atrial; **LVH**: left ventricular hypertrophy; **MI**: myocardial infarction; **PAD**: peripheral arterial disease; **T2D**: type 2 diabetes; **TIA**: transient ischemic attack**.**

Baseline characteristics of the the study population were calculated as **weighted means** according to the sample size of each trial arm.

62 **Table S2. Cochrane Risk of Bias tool (ROB)**

RCT	publishing year	Random sequence generation	Allocation concealment	Blinding of participant and staff	Blinding of outcome assessment	Incomplete outcome data	Selective reporting	Other bias
AASK	2002	LOW	LOW	MODERATE	LOW	LOW	LOW	LOW
		LOW	LOW	MODERATE	LOW	LOW	LOW	LOW
ALLHAT	2002	LOW	MODERATE	LOW	LOW	LOW	LOW	LOW
		LOW	MODERATE	LOW	LOW	LOW	LOW	LOW
AMADEO	2008	LOW	LOW	LOW	LOW	LOW	LOW	LOW
CONSENSUS II	1992	LOW	LOW	LOW	LOW	LOW	LOW	LOW
DIABHYCAR	2004	LOW	LOW	LOW	LOW	LOW	LOW	LOW
EUROPA	2003	LOW	LOW	LOW	LOW	LOW	LOW	LOW
GISSIAF	2009	LOW	LOW	LOW	LOW	LOW	LOW	LOW
HOPE	2000	LOW	LOW	LOW	LOW	LOW	LOW	LOW
IDNT	2001	LOW	LOW	LOW	LOW	LOW	LOW	LOW
		LOW	LOW	LOW	LOW	LOW	LOW	LOW
IMAGINE	2008	LOW	LOW	LOW	LOW	LOW	LOW	LOW
Kondo et al., 2003	2003	LOW	MODERATE	MODERATE	MODERATE	LOW	LOW	LOW
LIFE	2002	LOW	LOW	LOW	LOW	LOW	LOW	LOW
NAVIGATOR	2010	LOW	LOW	LOW	LOW	LOW	LOW	LOW
OLIVUS-Ex	2012	LOW	MODERATE	LOW	LOW	MODERATE	MODERATE	LOW
ONTARGET	2008	LOW	LOW	LOW	LOW	LOW	LOW	LOW
OPTIMAAL	2002	LOW	LOW	LOW	LOW	LOW	LOW	LOW
PART-2	2000	LOW	LOW	LOW	LOW	LOW	LOW	LOW
PREAMI	2006	LOW	LOW	LOW	LOW	LOW	LOW	LOW
PROGRESS	2001	LOW	LOW	LOW	LOW	LOW	LOW	LOW
PRoFESS	2008	LOW	LOW	LOW	LOW	LOW	LOW	LOW
QUIET	2001	LOW	LOW	LOW	LOW	LOW	LOW	LOW
ROADMAP	2011	LOW	LOW	LOW	LOW	LOW	LOW	LOW
SCAT	2000	LOW	LOW	LOW	LOW	LOW	LOW	LOW
SCOPE	2003	LOW	LOW	LOW	LOW	LOW	LOW	LOW
TRANSCEND	2008	LOW	LOW	LOW	LOW	LOW	LOW	LOW
VALUE	2004	LOW	LOW	LOW	LOW	LOW	LOW	LOW
VIVALD	2008	LOW	LOW	LOW	LOW	LOW	LOW	LOW

The risk of bias for each included study was assessed using The Cochrane Risk of Bias 2 (RoB2) tool. Most studies (18 out of 19) were determined to have a low risk of bias across the key domains (Random sequence generation, Allocation concealment, Blinding, Incomplete outcome data, Selective reporting, Other bias). Studies deemed to have a high or unclear risk in critical areas were considered in sensitivity analyses.

Table S3. Egger's Regression Tests for Funnel Plot Asymmetry

**Outcome**	**Test Method**	**Test Statistic**	**p-value**
**MACE**	t-test (lm)	t = -1.4807	0.2192
**H**HF	z-test (mixed)	z = -1.6254	0.1648
**All-cause death**	z-test (mixed)	z = -1.6216	0.1591

The Egger's tests showed no statistically significant evidence of funnel plot asymmetry for the major outcomes, suggesting a low likelihood of substantial publication bias.

Table S4. Harmonized Definitions of MACE and Composite Endpoints Across Included Trials

RCT	Components of MACE / Composite Endpoints	Outcome Type	Follow-up (Years)
**AASK**	CV death, hospitalization for MI, stroke, revascularization procedures, HF	Secondary	4
**ALLHAT**	(1) Non-fatal MI and fatal MI; (1) Combined CHD (CHD death, non-fatal MI, coronary revascularization, hospitalized angina); (3) Combined CVD (CHD death, non-fatal MI, stroke, revascularization, HHF, PAD)	(1) Primary / (2,3) Secondary	4.9
**AMADEO**	Death	Secondary	1
**CONSENSUS II**	(1) Death (any cause); (2) Reinfarction	(1) Primary; (2) Secondary	2.1
**DIABHYCAR**	(1) CV death, non-fatal MI, stroke, HHF; (2) Individual components of primary; (3) All-cause death, MI, stroke, HF	(1) Primary; (2,3) Secondary	3.92
**EUROPA**	(1) CV death, non-fatal MI, cardiac arrest; (2) Total mortality, non-fatal MI, hospitalization for unstable angina, cardiac arrest	(1) Primary; (2) Secondary	4.2
**GISSIAF**	(1) Hospitalization for a cardiovascular event; (2) Death	Secondary	1
**HOPE**	(1) MI, stroke, or CV death; (2) Individual components; (3) All-cause death	(1) Primary; (2,3) Secondary	4.5
**IDNT**	Death (any cause)	Primary	2.6
**IMAGINE**	(1) CV death, resuscitated cardiac arrest, non-fatal MI, coronary revascularization, hospitalization for angina, stroke, CHF; (2) Death (any cause)	(1) Primary; (2) Secondary;	2.95
**Kondo et al.**	(1) Non-fatal MI, revascularization, or CV death; (2) HHF	(1) Primary; (2) Secondary	2
**LIFE**	(1) CV death, MI, and stroke; (2) Individual components	(1) Primary; (2) Secondary	4.8

RCT	Components of MACE / Composite Endpoints	Outcome Type	Follow-up (Years)
**NAVIGATOR**	CV death, non-fatal MI, non-fatal stroke, HHF	Primary	5
**OLIVUS-Ex**	Cardiac or cerebral death, MI, stroke, rehospitalization due to unstable angina	Primary	3.41
**ONTARGET**	(1) CV death, HHF, MI, or stroke; (2) CV death, MI, or stroke	(1) Primary; (2,3) Secondary	4.6
**OPTIMAAL**	(1) All-cause death; (2) Fatal or non-fatal infarction	(1) Primary; (2) Tertiary	2.7
**PART-2**	(1) CHD death or non-fatal MI; (2) MI; (3) Stroke; (4) CHF	Secondary	4.7
**PREAMI**	Death, HHF	Primary	1
**PROGRESS**	(1) Fatal or non-fatal stroke; (2) Non-fatal stroke, non-fatal MI, or death (any vascular cause)	(1) Primary; (2) Secondary	3.9
**PRoFESS**	(1) Stroke; (2) CV death, MI, stroke, or worsening/new HF	(1) Primary; (2) Secondary	2.5
**QUIET**	(1) Cardiac death, MI, revascularization; (2) Cardiac death, non-fatal MI, or resuscitated cardiac arrest	(1) Primary; (2) Secondary	2.25
**ROADMAP**	(1) All-cause death; (2) CV death	Secondary	4
**SCAT**	Death, MI, stroke, revascularization, or hospitalization from angina	Primary	3.98
**SCOPE**	(1) CV death, non-fatal MI, non-fatal stroke; (2) Individual components	(1) Primary; (2) Secondary	3.7
**TRANSCEND**	(1) CV death, MI, stroke, or HHF; (2) CV death, MI, stroke	(1) Primary; (2) Secondary	4.6

RCT	Components of MACE / Composite Endpoints	Outcome Type	Follow-up (Years)
**VALUE**	(1) Sudden cardiac death, fatal MI, death during/after PCI, non-fatal MI, HHF, emergency MI procedures; (2) Fatal/non-fatal MI, HF, and stroke	(1) Primary; (2,3) Secondary	4.2
**VIVALD**	(1) All-cause death; (2) MI, stroke	Secondary	1

CV = Cardiovascular; CHD = Coronary Heart Disease; CVD = Cardiovascular Disease; HF = Heart Failure; HHF = Hospitalization for Heart Failure; MI = Myocardial Infarction; UA = Unstable Angina; PAD = Peripheral Artery Disease.

Table S5. GRADE Summary of Findings for Network Meta-Analysis

Outcome	Comparison (Active vs. Placebo)	No. of Studies (Patients)	Certainty of Evidence (GRADE)	Reasons for Downgrading
**MACE**	Candesartan	2	**High**	None. Low risk of bias and consistent direct/indirect evidence.
	Telmisartan	2	**High**	None. Results consistent across sensitivity analyses.
	Others (Losartan, Valsartan, etc.)	Varies	**Moderate**	Downgraded 1 level for imprecision (wide 95% CI).
**H**HF	Irbesartan	1	**Moderate**	Downgraded 1 level for imprecision (limited direct comparisons).
	Others	Varies	**Moderate**	Downgraded 1 level for imprecision.
**All-cause Death**	All ARBs	27	**High**	Low inconsistency (I^2=38%) and low risk of bias.

Figure S1. Flow diagram

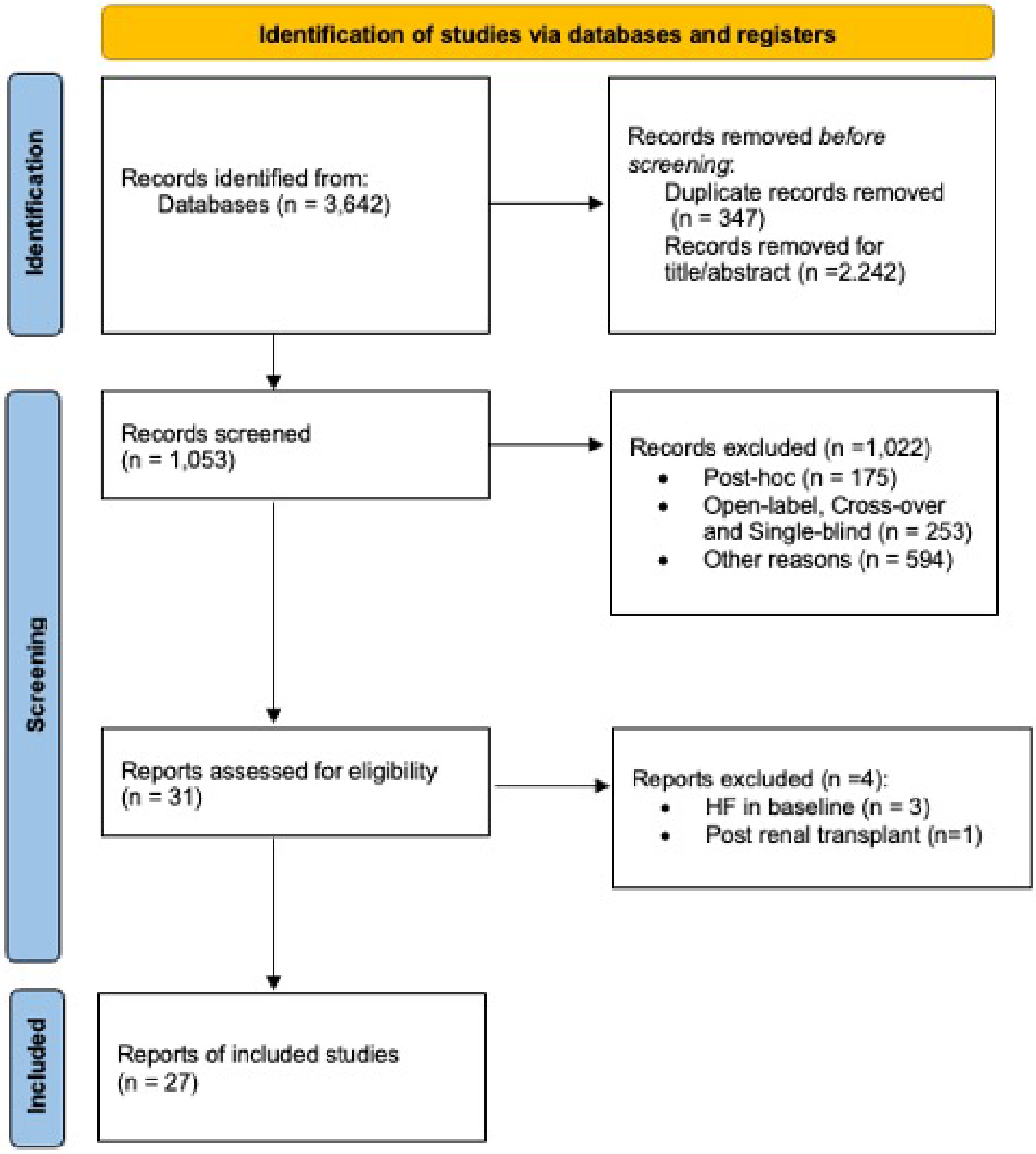

Figure S2. Study Networks: A plot illustrating the network of comparisons between different antihypertensive treatments for primary and secondary outcomes

a. MACE

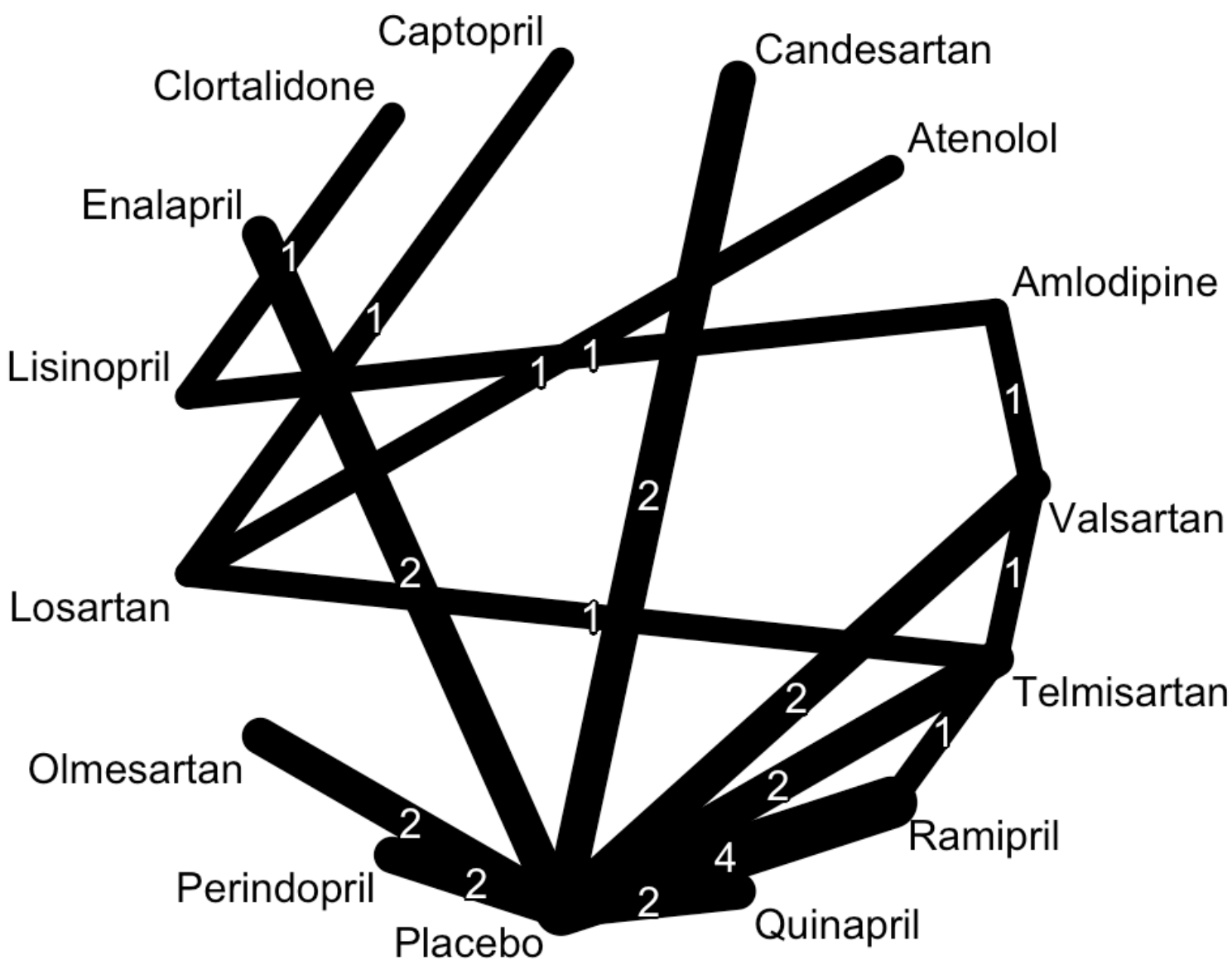

b. HHF

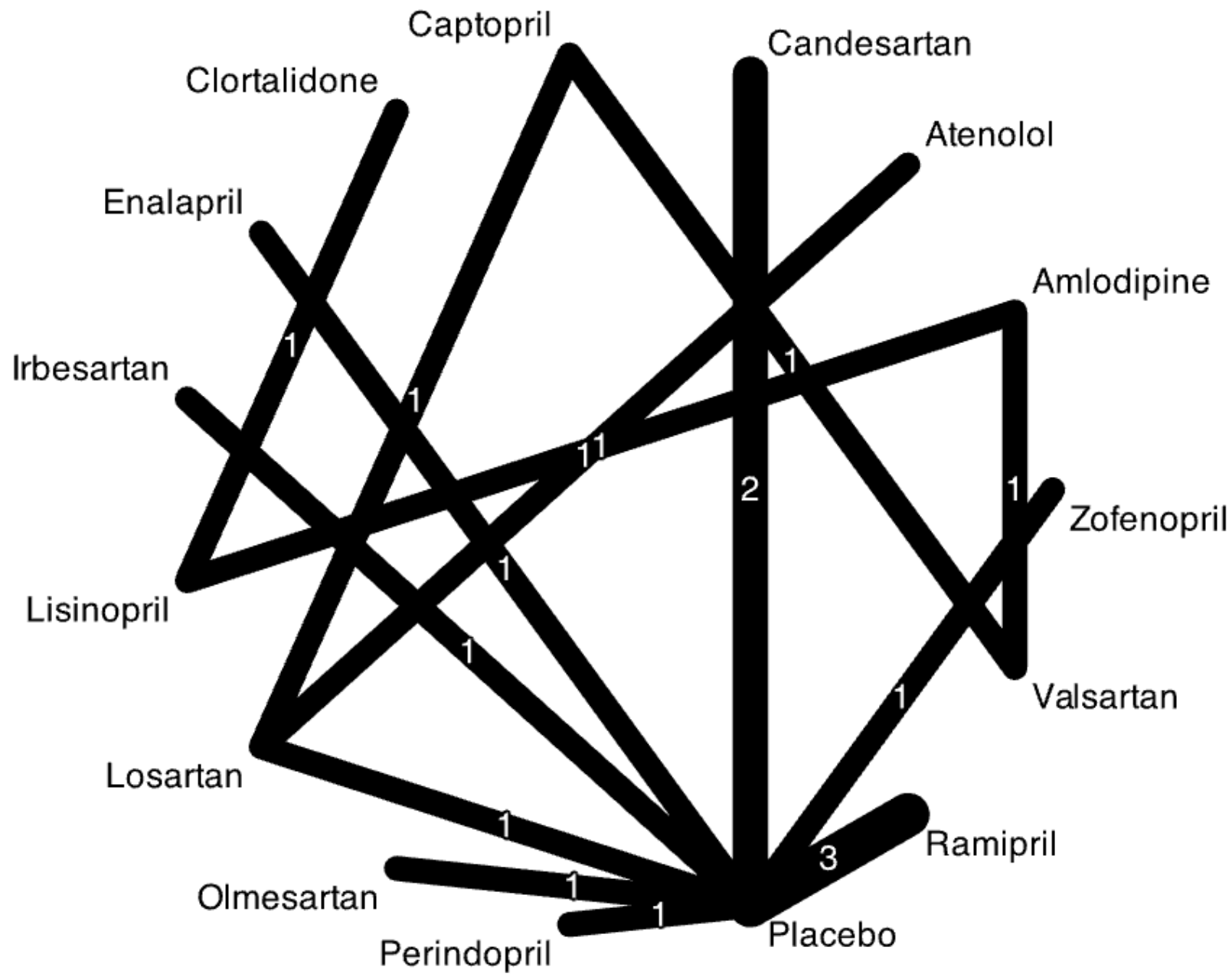

Captopril
Candesartan
Clortalidone
Atenolol
Enalapril
Amlodipine
Irbesartan
Zofenopril
Lisinopril
Valsartan
Losartan
Ramipril
Olmesartan
Perindopril
Placebo
1
1
1
11
2
1
1
1
1
1
3
1
1

c. All-cause death

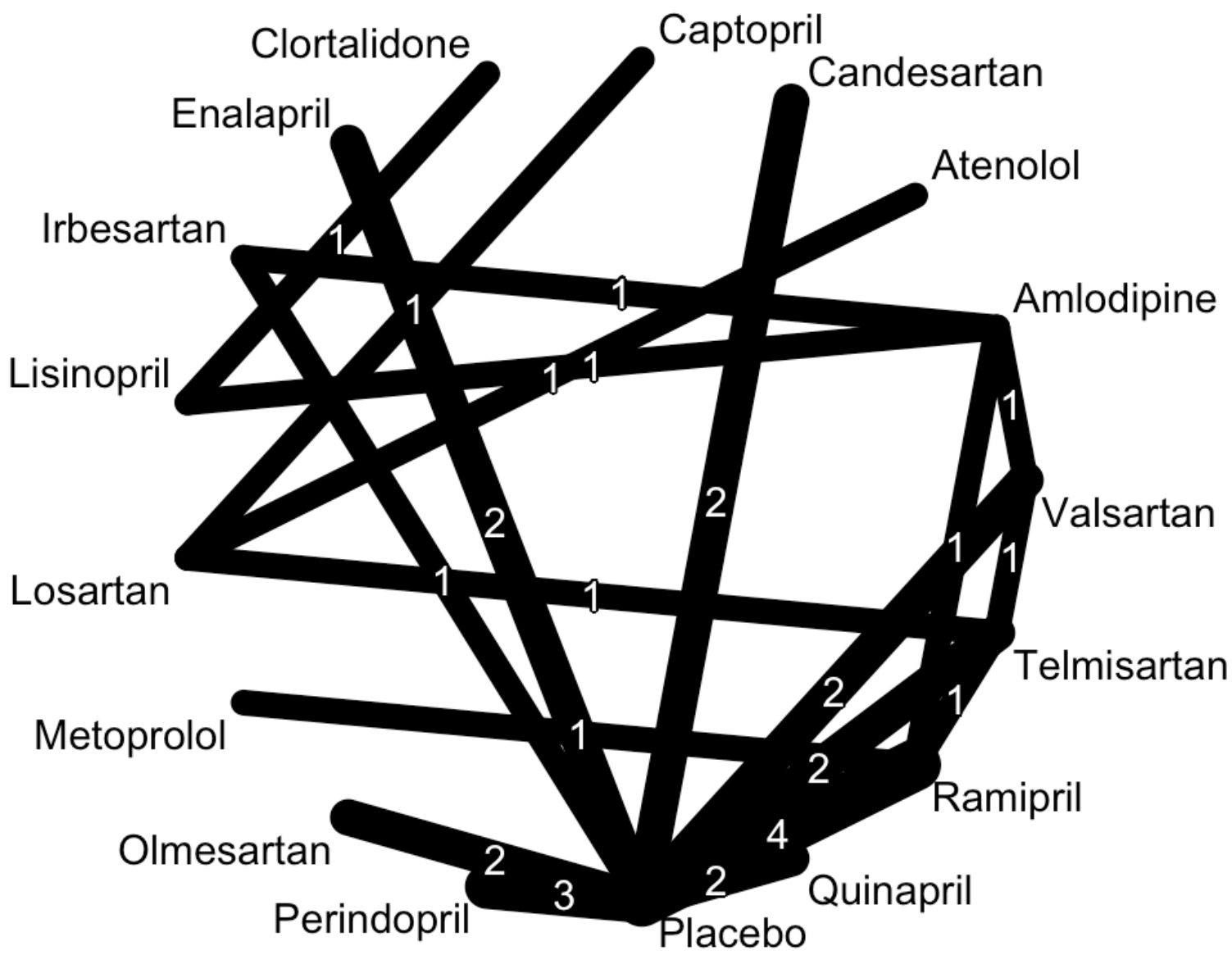

Figure S3. Funnel Plots: Funnel plots were generated for MACE, HHF, and All-cause death to visually assess publication bias.

a. MACE

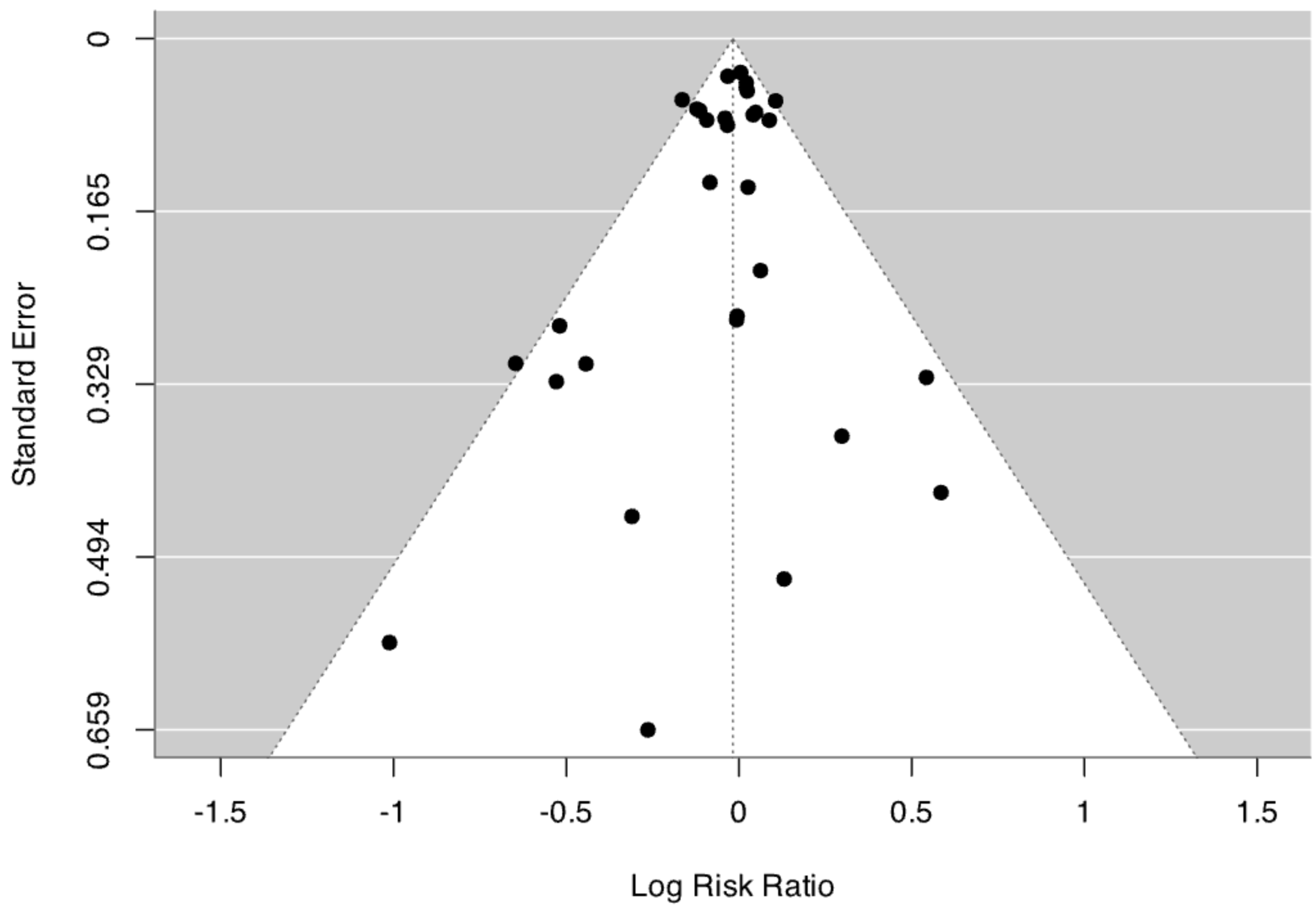

b. HHF

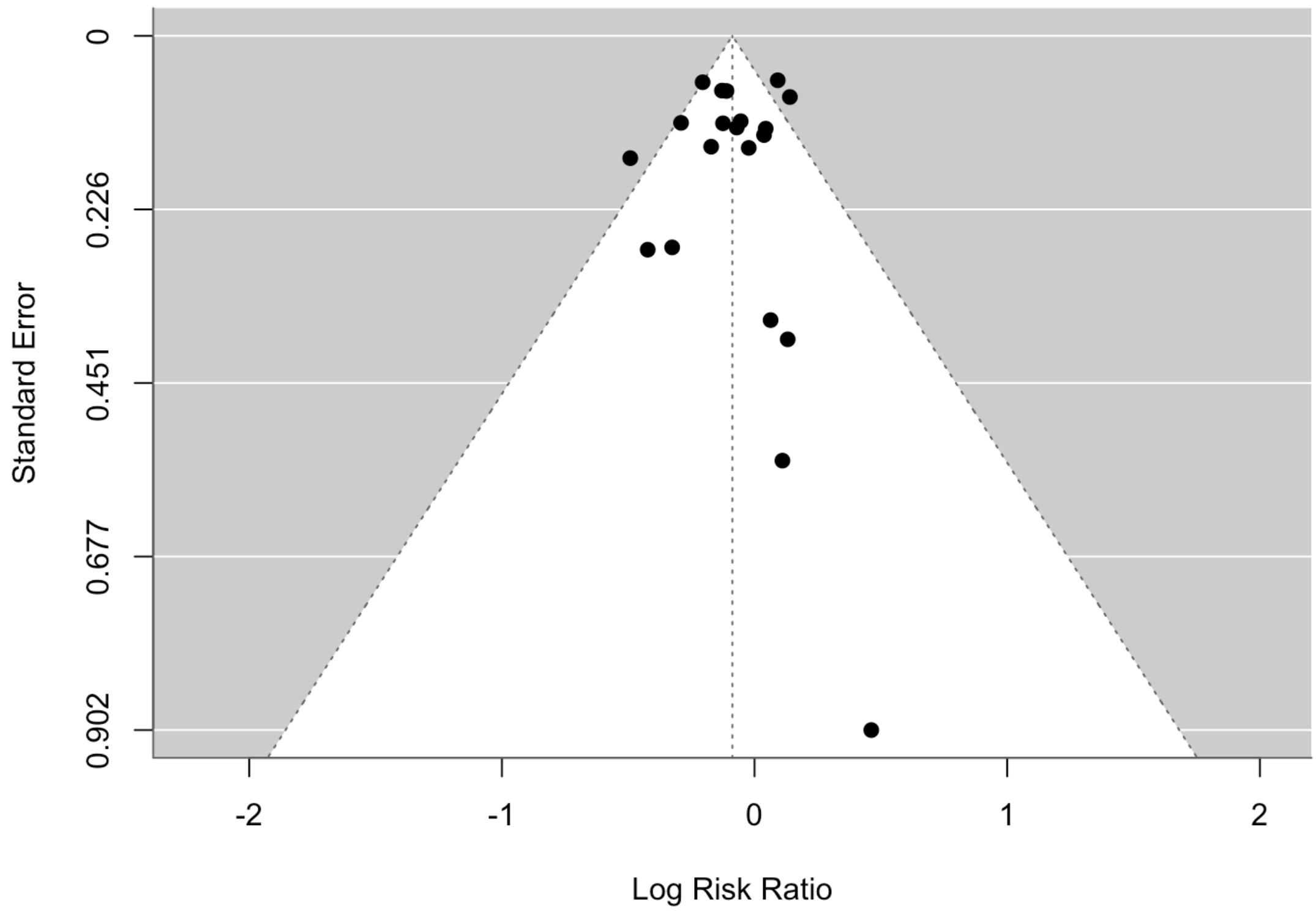

c. All-cause death

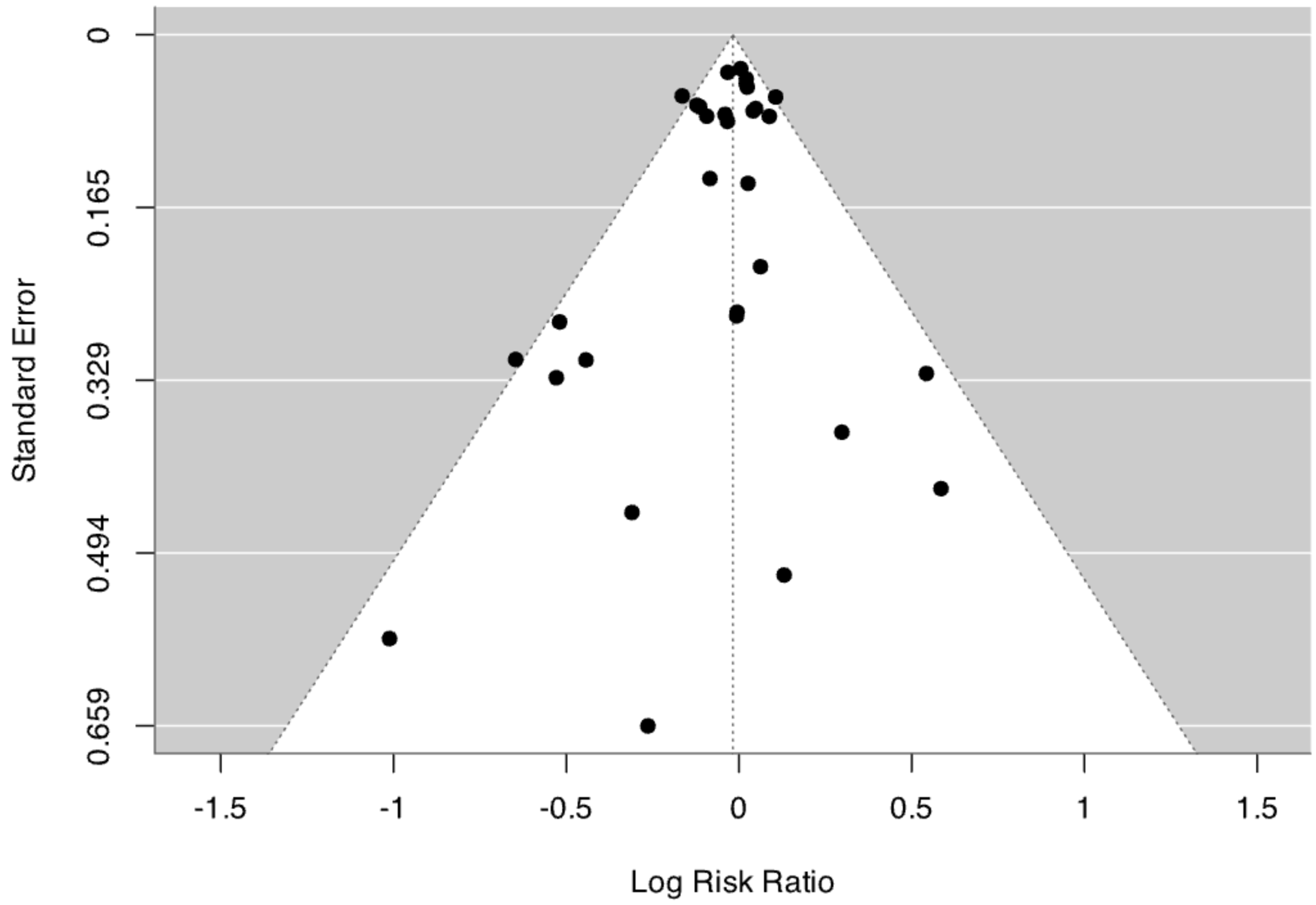

Figure S4. Forest Plots for Sensitivity Analyses Primary and Secondary Outcomes: Detailed Forest plots for MI, Stroke, CV Death, MACE and All-cause Death considering the exclusion of 25% of studies in a random basis.

a. MACE

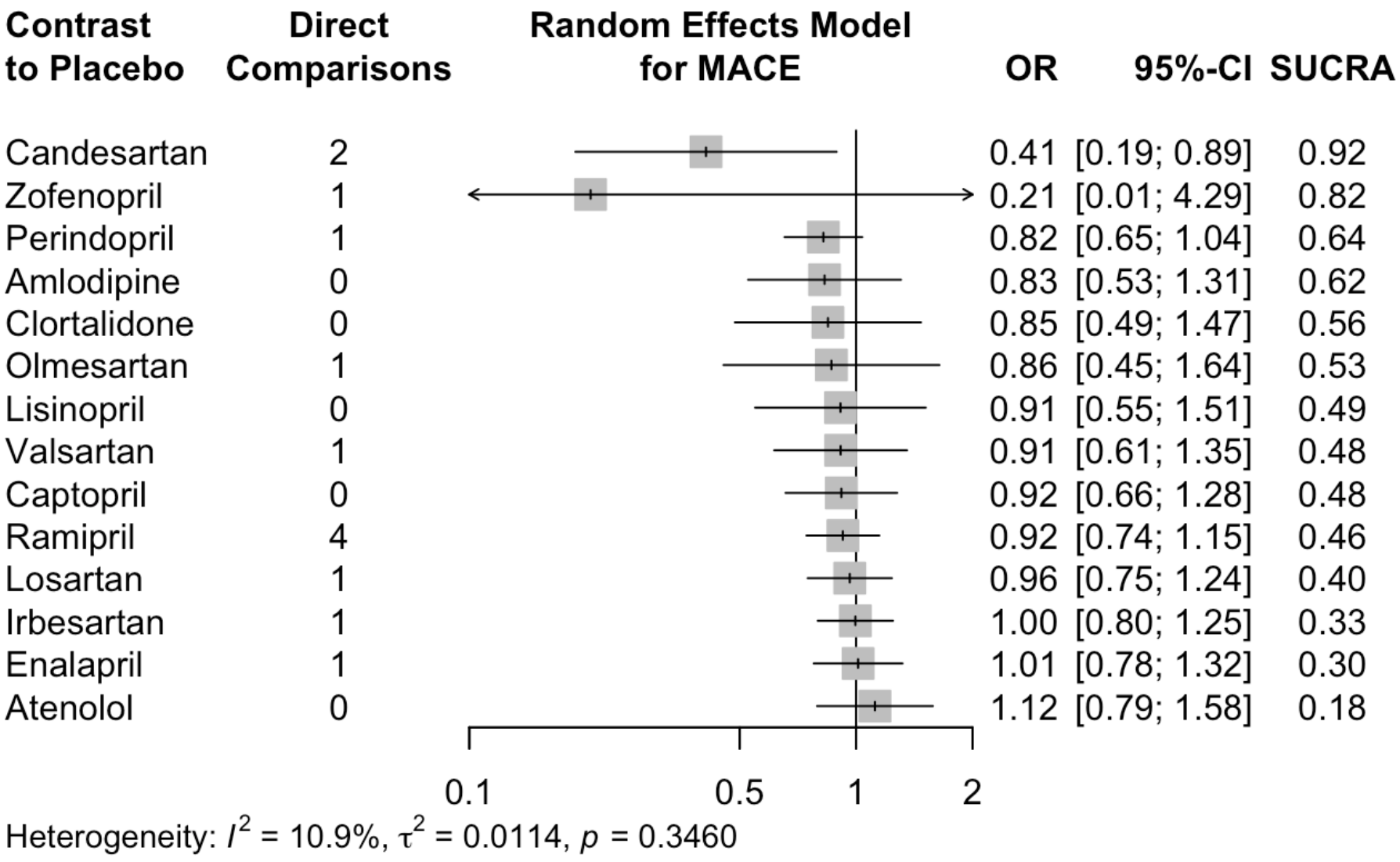

b. HHF

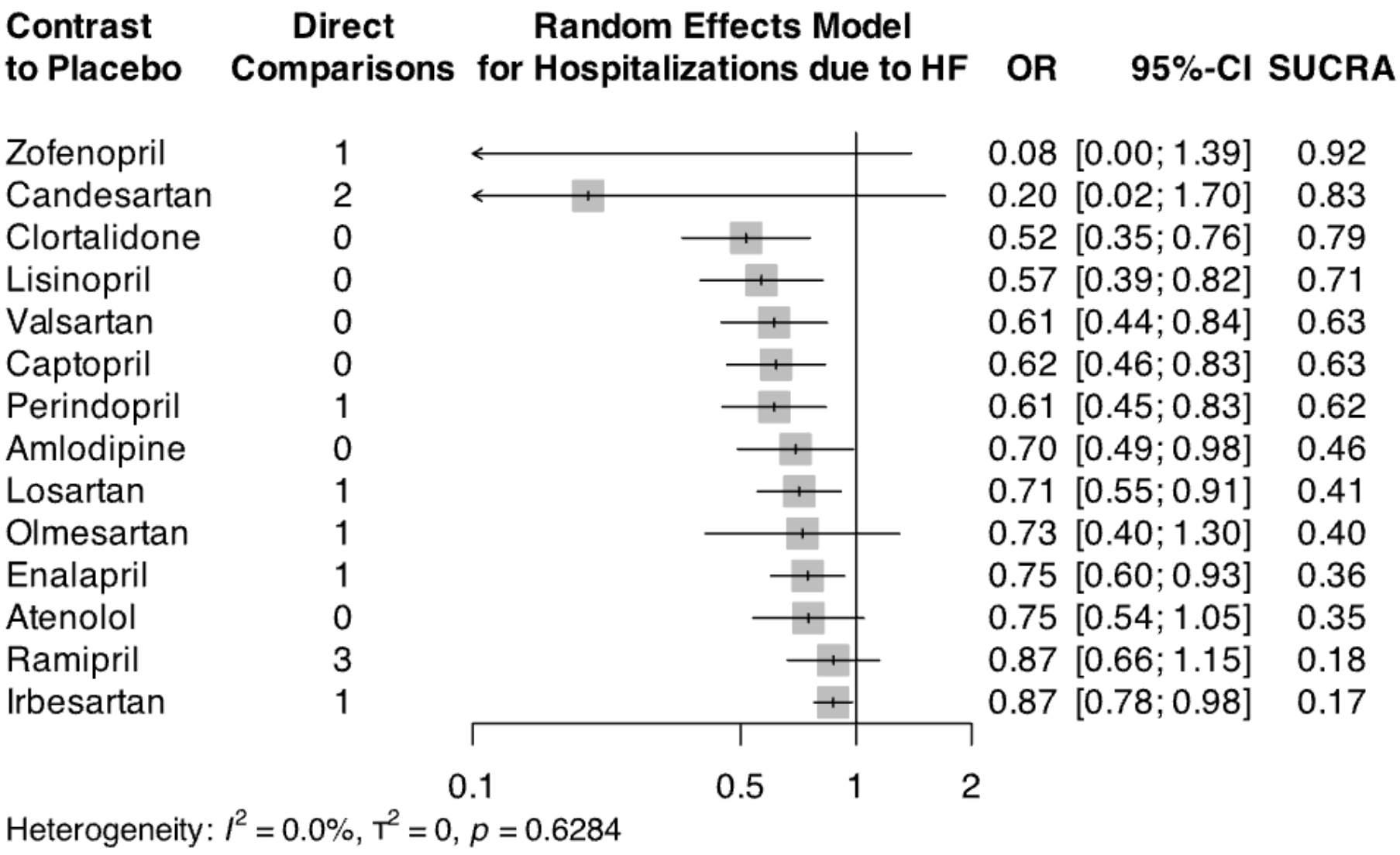

c. All-cause death

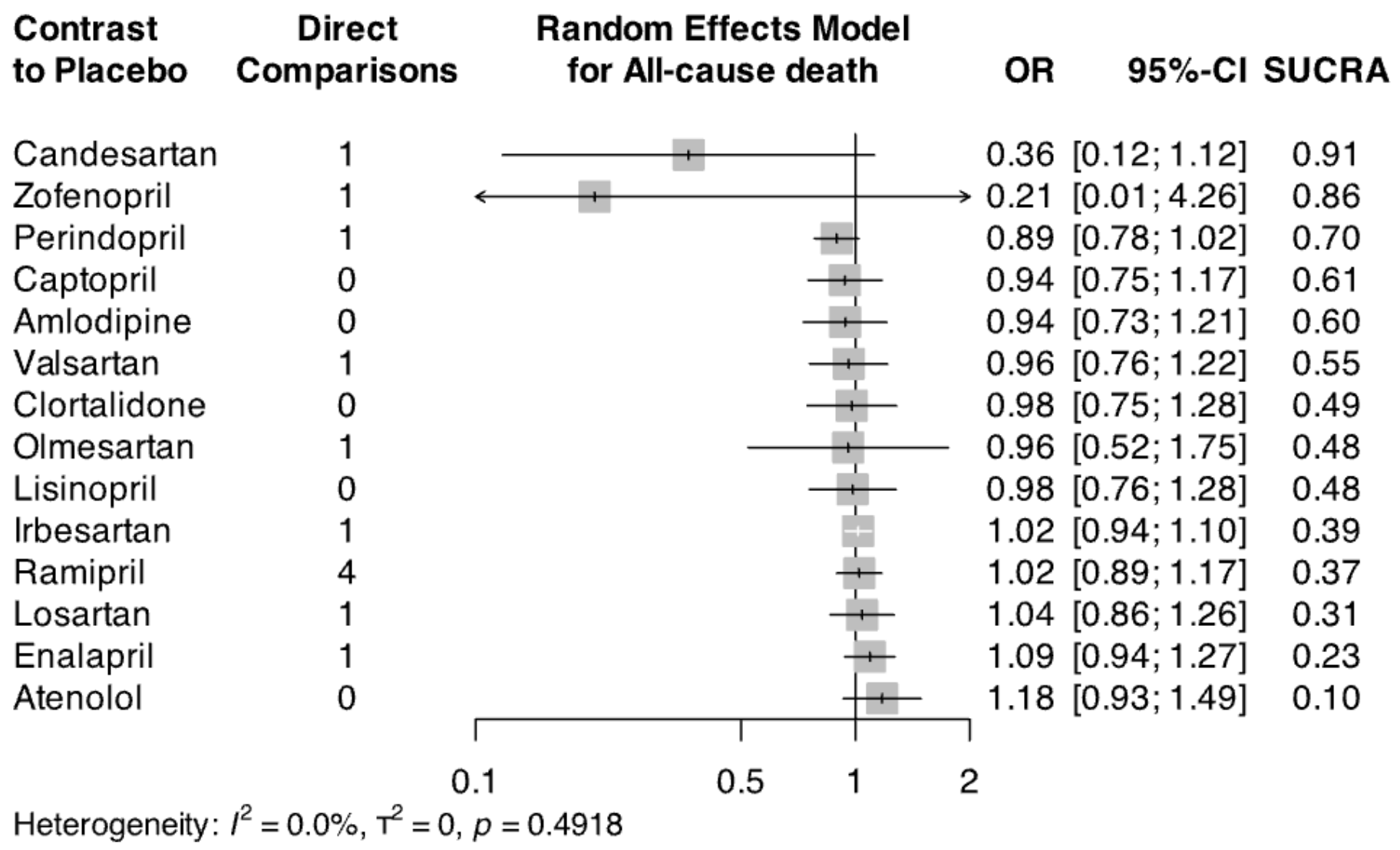

Figure S5. Forest Plots including both ARBs and non-ARBs antihypertensive drugs used for the indirect comparisons for Primary and Secondary Outcomes

a. MACE

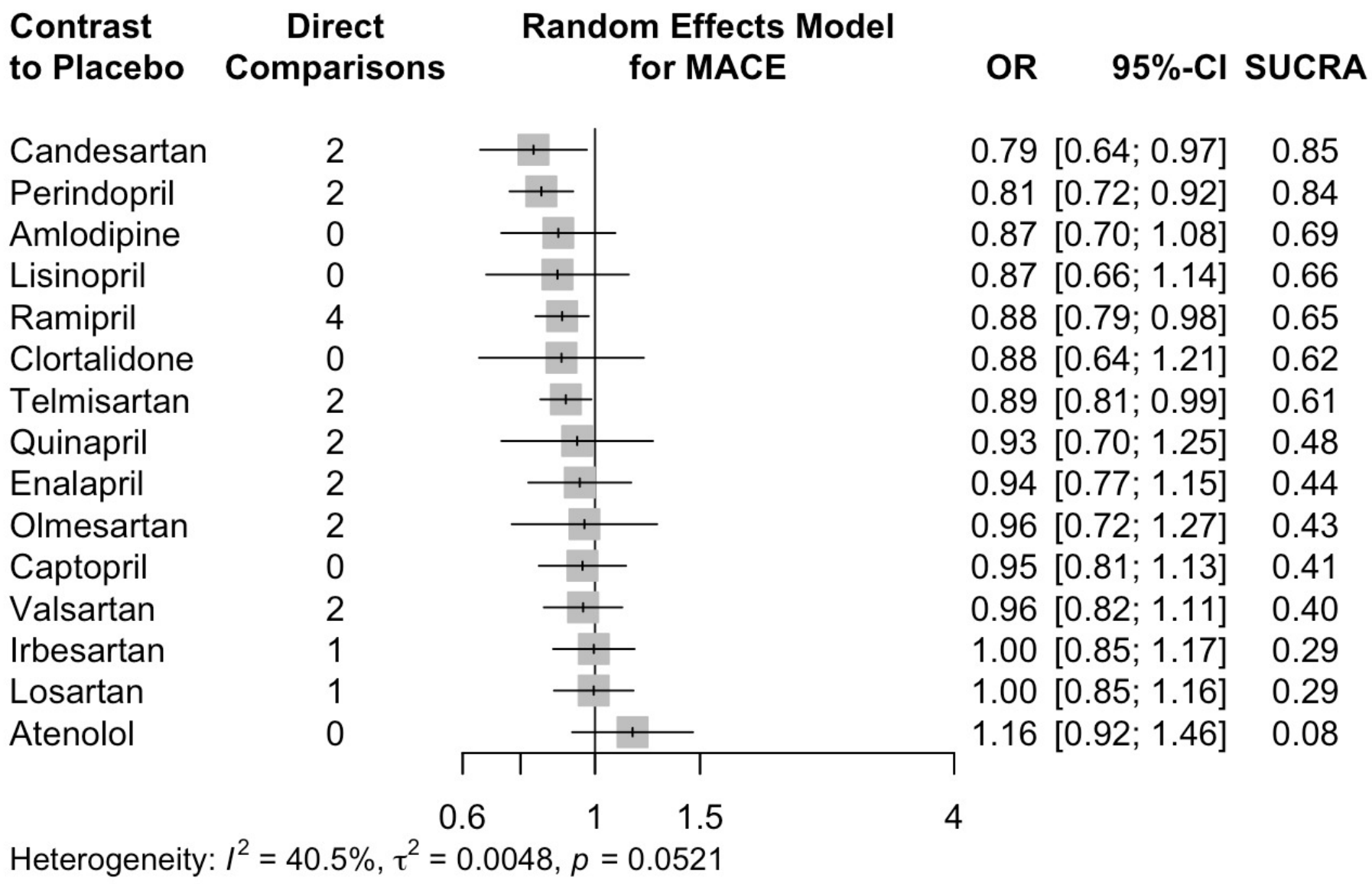

b. HHF

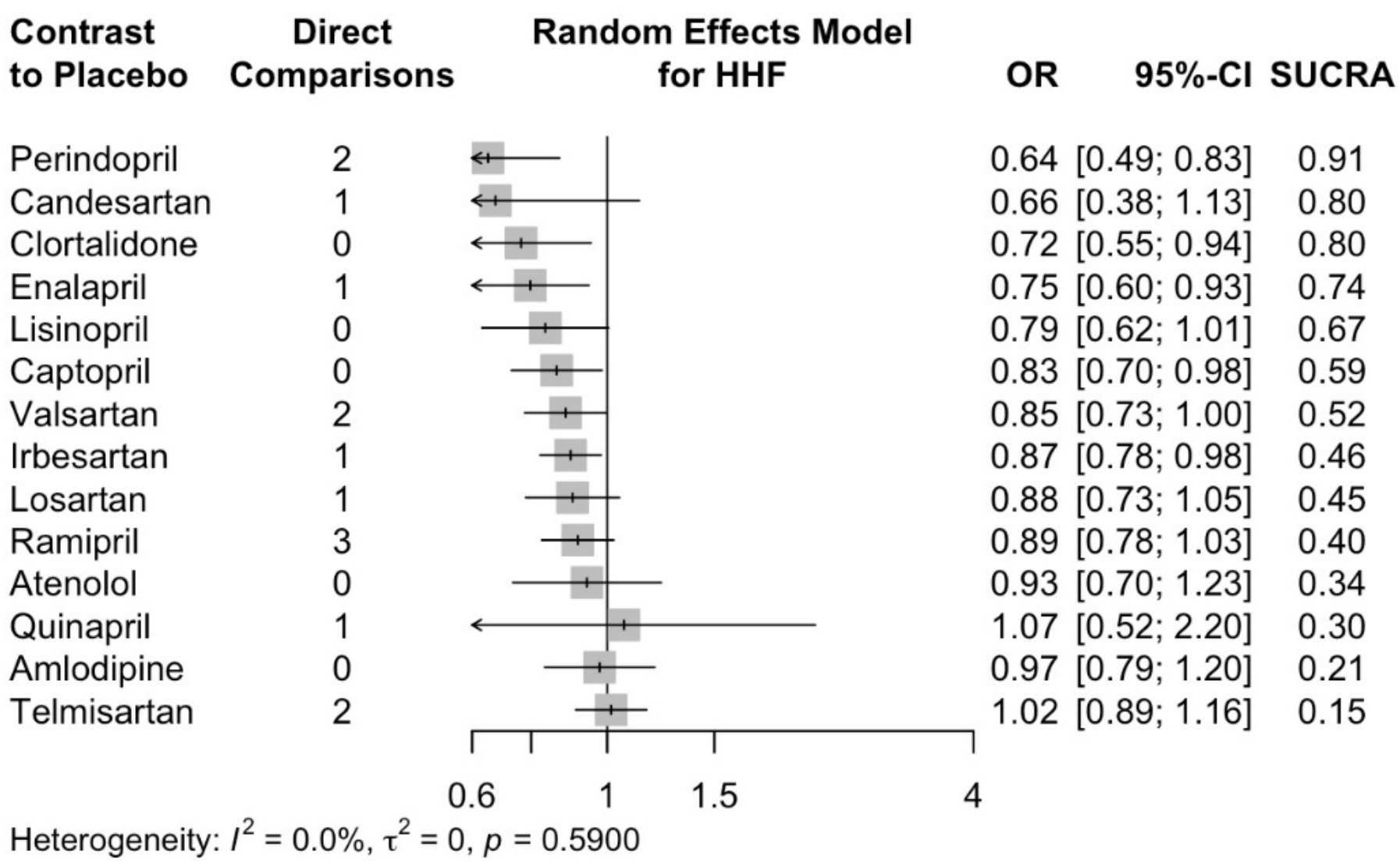

c. All-cause death

Contrast to Placebo	Direct Comparisons	Random Effects Model for All-cause death	OR	95%-CI	SUCRA
Lisinopril	0		0.93	[0.70; 1.23]	0.70
Ramipril	3		0.93	[0.83; 1.05]	0.70
Perindopril	3		0.94	[0.81; 1.08]	0.70
Candesartan	2		0.93	[0.75; 1.16]	0.70
Clortalidone	0		0.93	[0.67; 1.29]	0.69
Valsartan	2		0.94	[0.78; 1.13]	0.68
Irbesartan	1		0.95	[0.74; 1.22]	0.66
Amlodipine	0		0.96	[0.77; 1.20]	0.66
Quinapril	2		0.99	[0.68; 1.46]	0.59
Telmisartan	2		1.00	[0.89; 1.12]	0.56
Enalapril	2		1.07	[0.86; 1.32]	0.46
Olmesartan	2		1.46	[0.82; 2.62]	0.23
Captopril	0		1.52	[0.76; 3.06]	0.20
Metoprolol	0		1.57	[0.89; 2.78]	0.18
Losartan	0		1.69	[0.86; 3.31]	0.14
Atenolol	0		1.91	[0.95; 3.85]	0.10

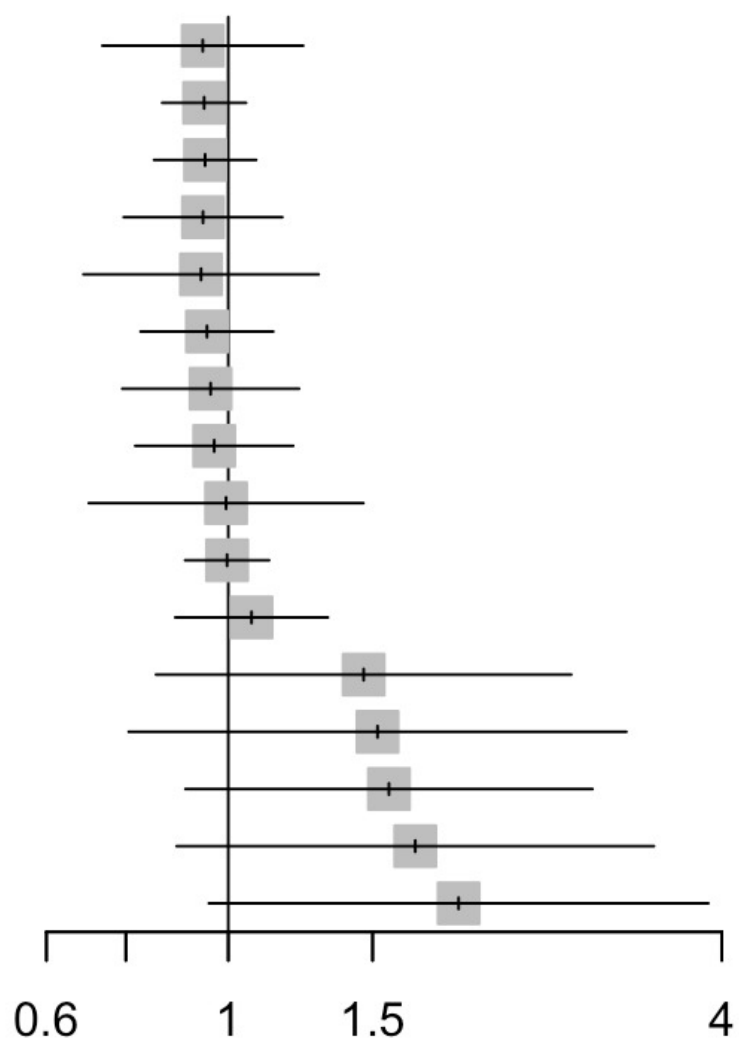

Heterogeneity: I^2 = 37.8%, τ^2 = 0.0066, p = 0.0691

Figure S6. Forest Plots excluding RCTs that considered ACEi vs placebo for Primary and Secondary Outcomes

a. MACE

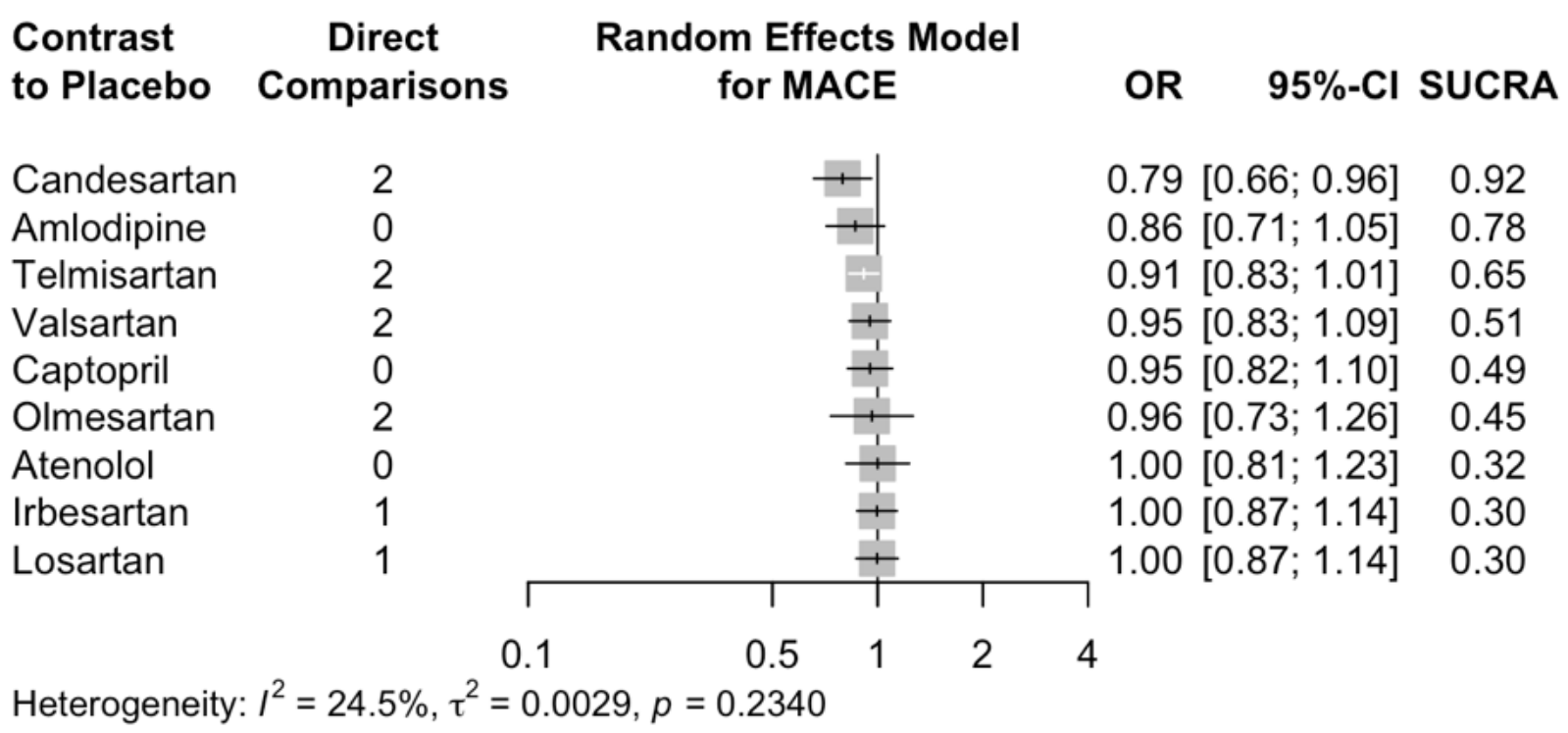

b. HHF

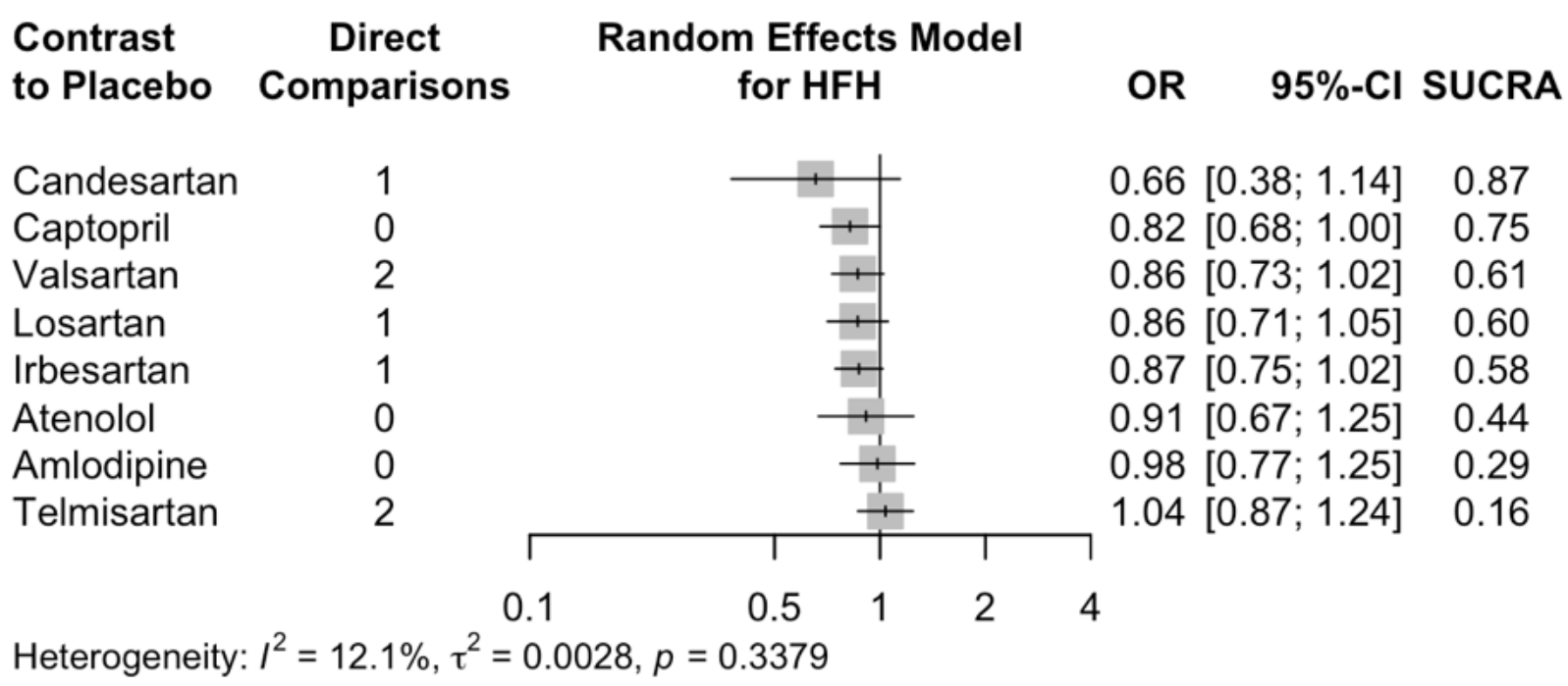

c. All-cause death

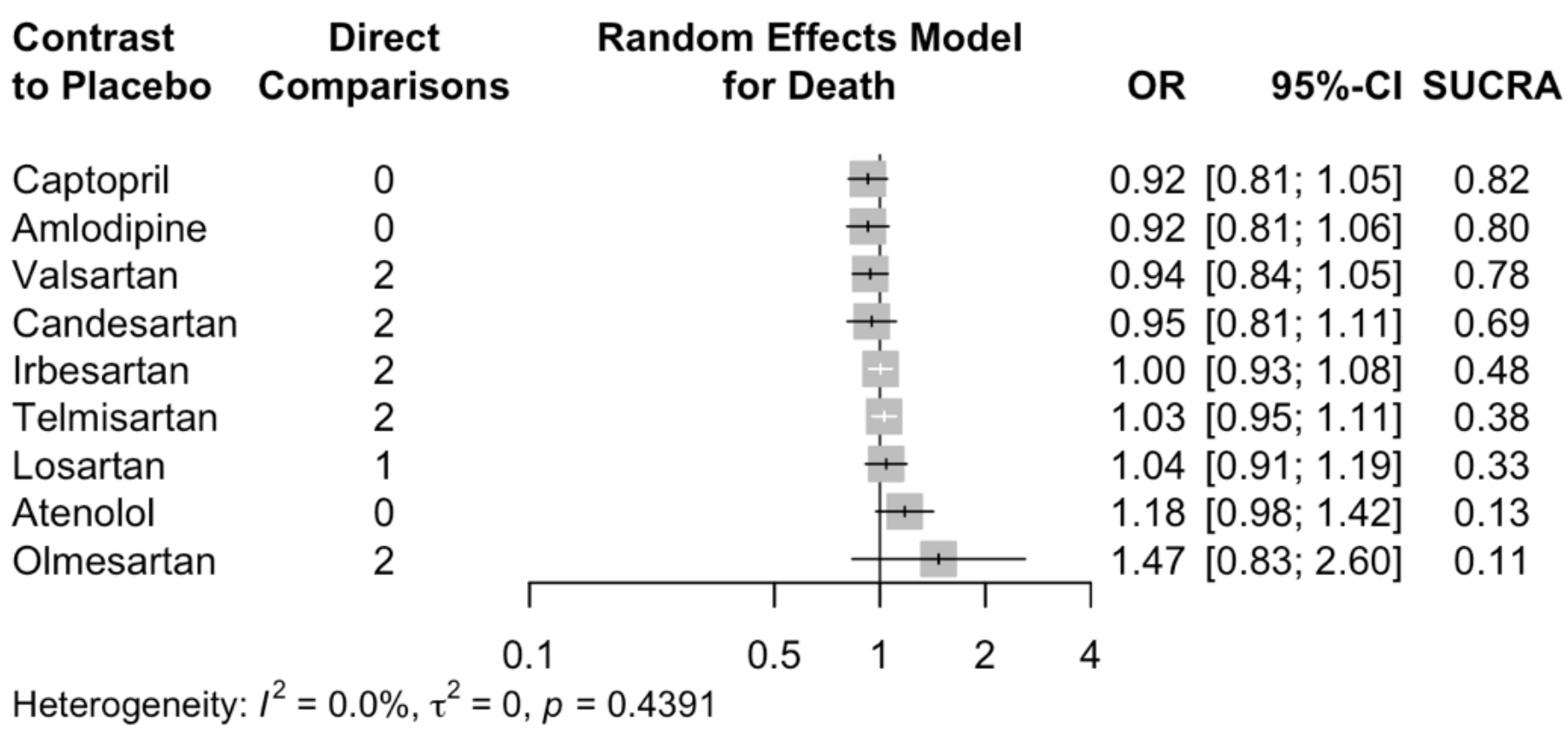

Figure S7. Meta-regression plots for all-cause death and MACE

a. MACE

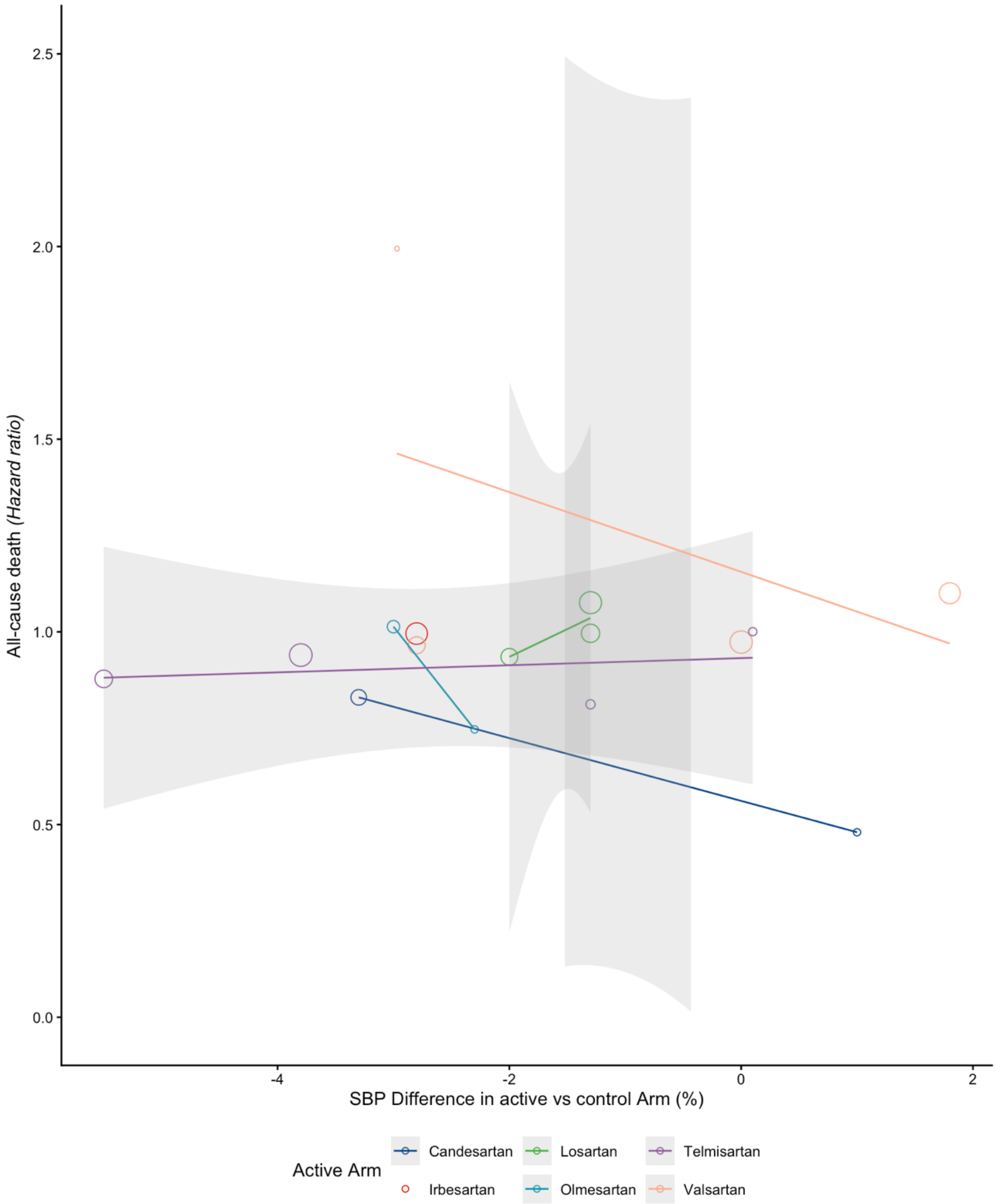

b. All-cause death

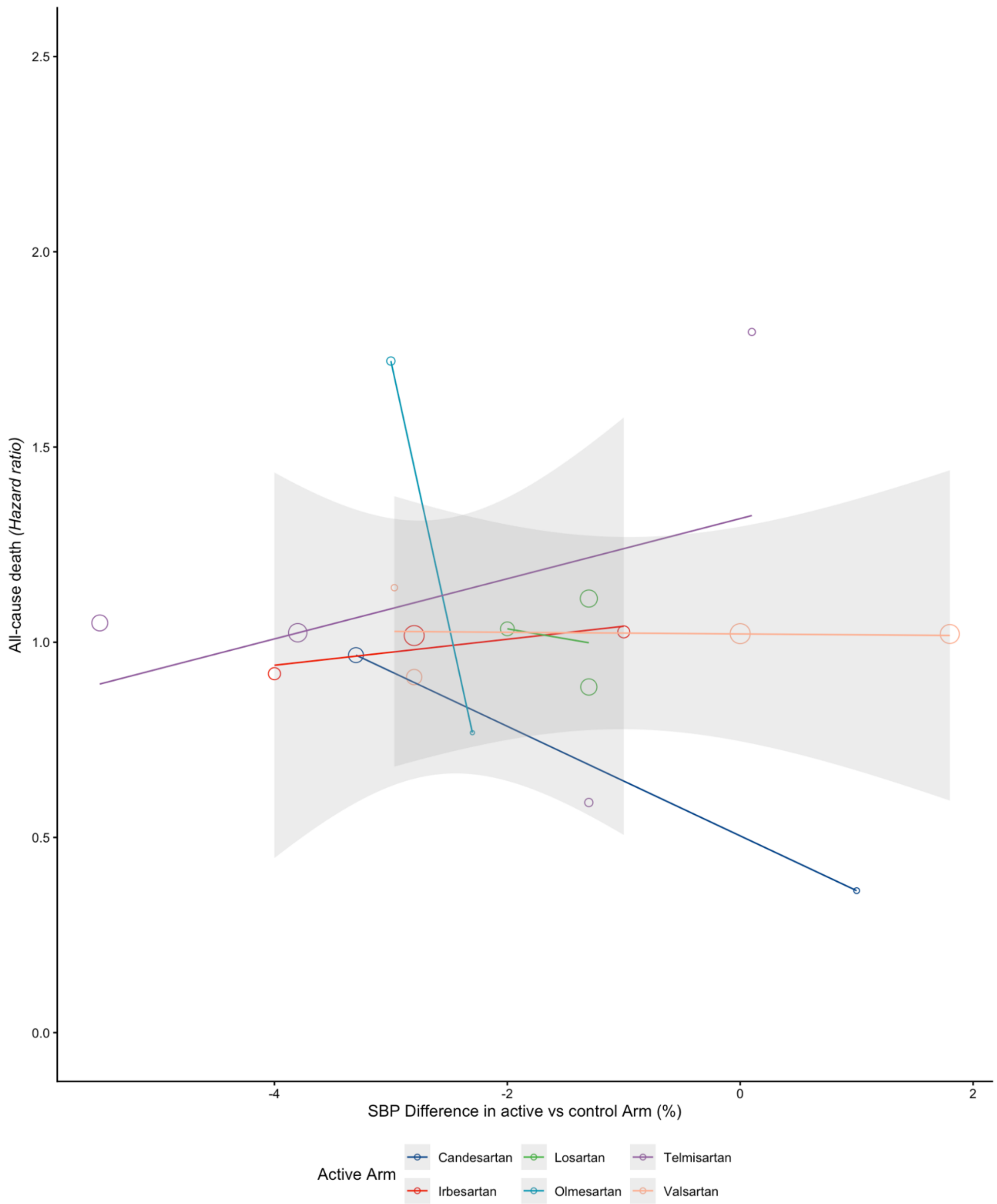

Figure S8. Forest plots stratified by the degree of SBP change (difference between active and control groups higher than -1.3mmHg, between -1.3 to -2.9mmHg, and lower than -2.9mmHg)

a. MACE

Name	Active	Control	HR [95% CI]
LIFE	508/4605	508/4588	1.00 [0.89, 1.12]
OPTIMAAL	944/2744	874/2733	1.08 [1.00, 1.16]
VALIANT	1102/4909	1132/4909	0.97 [0.91, 1.05]
VALUE	995/7649	898/7596	1.10 [1.01, 1.20]
VIVALD	23/443	22/424	1.00 [0.57, 1.77]
Kondo et al., 2003	12/203	25/203	0.48 [0.25, 0.93]
Diff change in SBP > -1.3mmHg	3584/20553459/2045		1.03 [0.99, 1.08]
ACTIVE-I	963/4518	963/4498	1.00 [0.92, 1.08]
AMADEO	27/419	35/441	0.81 [0.50, 1.32]
NAVIGATOR	371/4631	388/4675	0.97 [0.84, 1.11]
OLIVUS-Ex	14/126	18/121	0.75 [0.39, 1.43]
RENAAL	247/751	268/762	0.94 [0.81, 1.08]
Diff change in SBP between -1.3 to -2.9mmHg	1622/10445672/1049		0.97 [0.91, 1.03]
GISSIAF	18/722	9/720	1.99 [0.90, 4.41]
PRoFESS	1289/10146377/1018		0.94 [0.88, 1.01]
ROADMAP	96/2232	94/2215	1.01 [0.77, 1.34]
SCOPE	224/2477	268/2460	0.83 [0.70, 0.98]
TRANSCEND	384/2954	440/2972	0.88 [0.77, 1.00]
Diff change in SBP < -2.9mmHg	2011/18532188/1855		0.92 [0.87, 0.97]

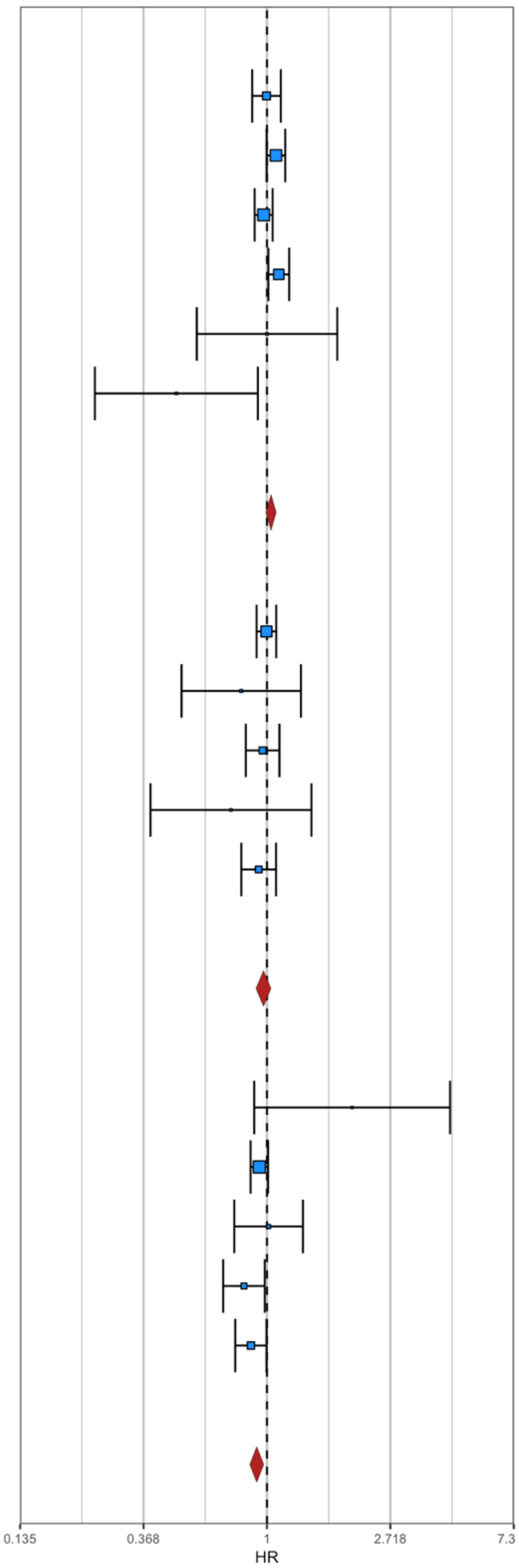

b. All-cause death

Name	Active	Control	HR [95% CI]
IDNT (2)	87/579	83/567	1.03 [0.78, 1.35]
LIFE	383/4605	431/4588	0.89 [0.78, 1.01]
OPTIMAAL	499/2744	447/2733	1.11 [0.99, 1.25]
VALIANT	979/4909	958/4909	1.02 [0.94, 1.11]
VALUE	841/7649	818/7596	1.02 [0.93, 1.12]
VIVALD	15/443	8/424	1.79 [0.77, 4.19]
Kondo et al., 2003	4/203	11/203	0.36 [0.12, 1.12]
Diff change in SBP > -1.3mmHg	2808/2113	2756/2102	1.02 [0.97, 1.07]
ACTIVE-I	949/4518	929/4498	1.02 [0.94, 1.10]
AMADEO	14/419	25/441	0.59 [0.31, 1.12]
NAVIGATOR	295/4631	327/4675	0.91 [0.78, 1.06]
OLIVUS-Ex	4/126	5/121	0.77 [0.21, 2.79]
RENAAL	158/751	155/762	1.03 [0.85, 1.26]
Diff change in SBP between -1.3 to -2.9mmHg	1420/1044	1441/1049	0.99 [0.93, 1.06]
GISSIAF	8/722	7/720	1.14 [0.42, 3.13]
IDNT (1)	87/579	93/569	0.92 [0.70, 1.20]
PRoFESS	755/10146	740/10186	1.02 [0.93, 1.13]
ROADMAP	26/2232	15/2215	1.72 [0.91, 3.24]
SCOPE	259/2477	266/2460	0.97 [0.82, 1.14]
TRANSCEND	364/2954	349/2972	1.05 [0.91, 1.20]
Diff change in SBP < -2.9mmHg	1499/1911	1470/1912	1.02 [0.95, 1.09]

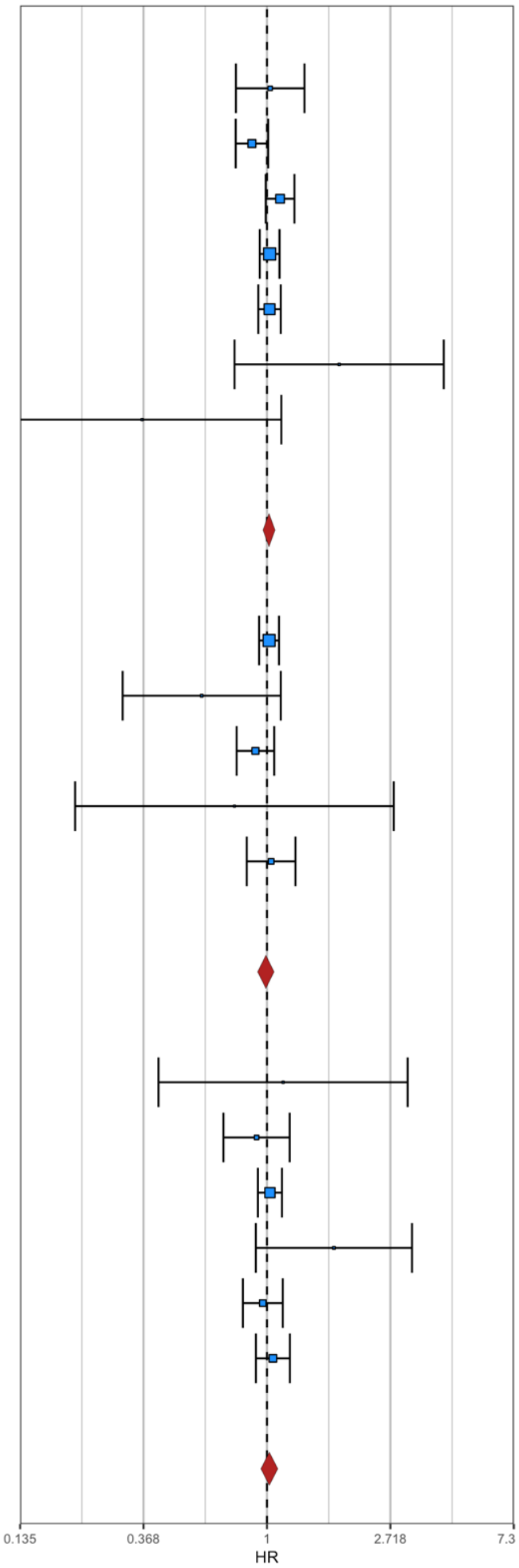

Figure S9. Rankograms showing the probability distribution of treatment rankings

a. MACE

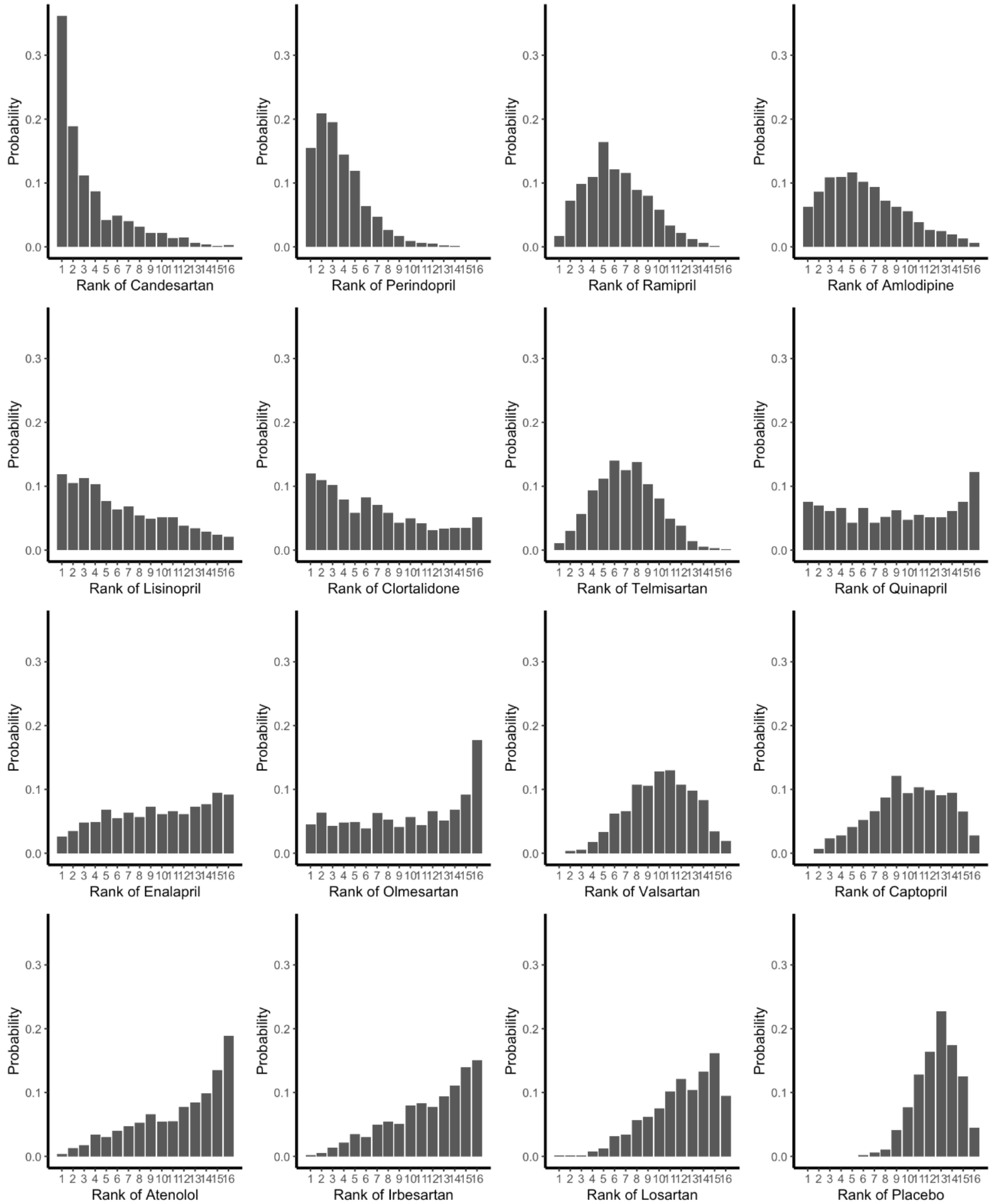

b. All-cause death

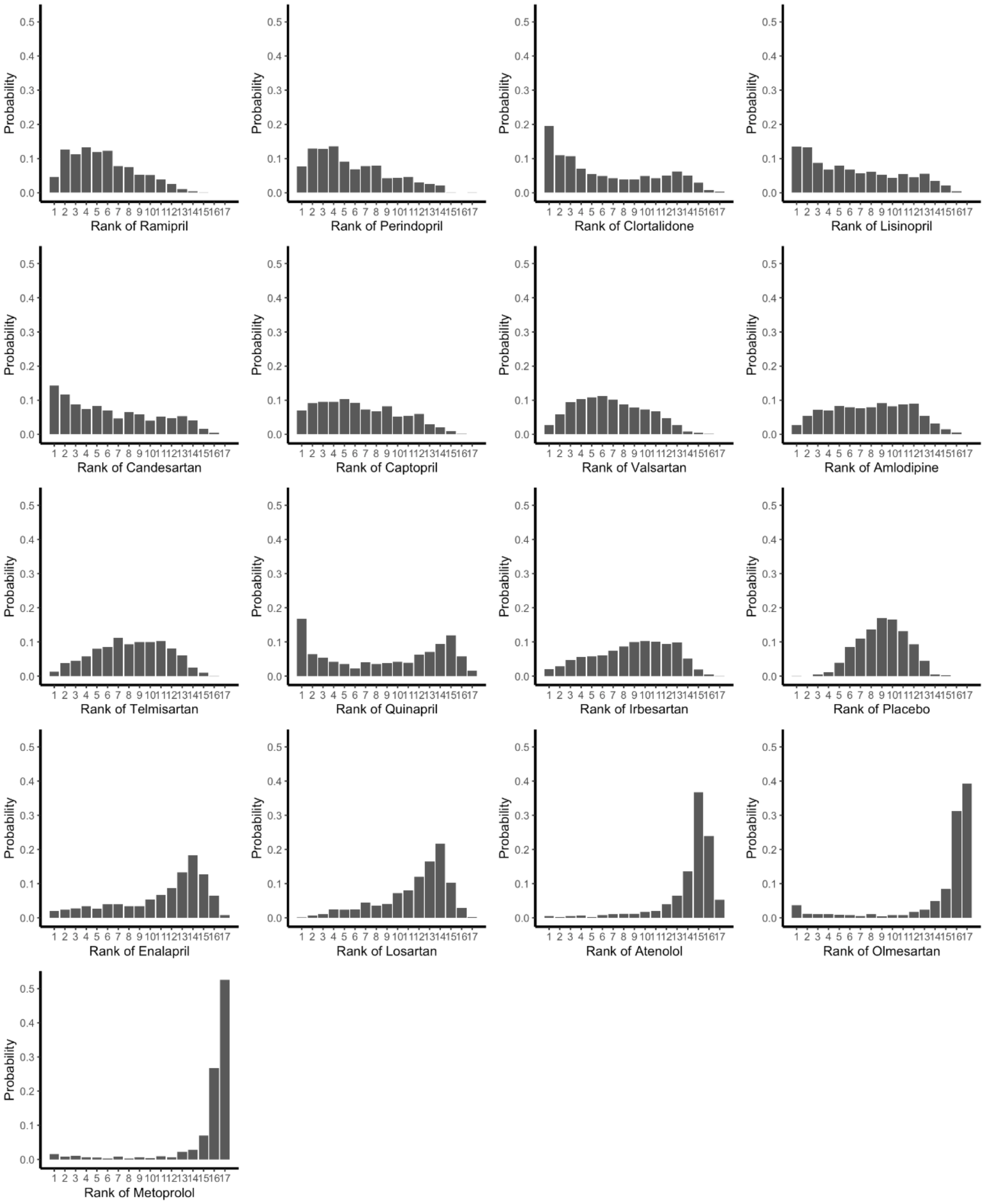

Table S6. League table of pairwise comparisons for MACE

Amlodipine	Atenolol	Candesartan	Captopril	Clortalidone	Enalapril	Irbesartan	Lisinopril	Losartan	Olmesartan	Perindopril
Amlodipine	.	.	.	.	.	.	1.00 (0.85 - 1.18)	.	.	.
0.87 (0.65 - 1.16)	Atenolol	.	.	.	.	.	.	1.00 (0.84 - 1.20)	.	.
1.10 (0.82 - 1.48)	1.27 (0.93 - 1.72)	Candesartan	.	.	.	.	.	.	.	.
0.91 (0.74 - 1.12)	1.05 (0.84 - 1.31)	0.83 (0.64 - 1.08)	Captopril	.	.	.	.	0.93 (0.80 - 1.08)	.	.
0.99 (0.79 - 1.24)	1.14 (0.79 - 1.64)	0.90 (0.62 - 1.31)	1.08 (0.80 - 1.47)	Clortalidone	.	.	1.02 (0.87 - 1.19)	.	.	.
0.92 (0.69 - 1.23)	1.06 (0.78 - 1.43)	0.84 (0.63 - 1.11)	1.01 (0.78 - 1.31)	0.93 (0.64 - 1.35)	Enalapril	.	.	.	.	.
0.87 (0.67 - 1.14)	1.00 (0.76 - 1.33)	0.79 (0.61 - 1.02)	0.96 (0.76 - 1.20)	0.88 (0.62 - 1.25)	0.95 (0.74 - 1.22)	Irbesartan	.	.	.	.
1.00 (0.85 - 1.18)	1.16 (0.83 - 1.61)	0.91 (0.65 - 1.28)	1.10 (0.85 - 1.43)	1.02 (0.87 - 1.19)	1.09 (0.78 - 1.53)	1.15 (0.84 - 1.57)	Lisinopril	.	.	.
0.87 (0.69 - 1.09)	1.00 (0.84 - 1.20)	0.79 (0.62 - 1.02)	0.96 (0.84 - 1.10)	0.88 (0.64 - 1.22)	0.95 (0.74 - 1.22)	1.00 (0.81 - 1.24)	0.87 (0.66 - 1.15)	Losartan	.	.
0.90 (0.63 - 1.29)	1.04 (0.72 - 1.50)	0.82 (0.58 - 1.16)	0.99 (0.72 - 1.37)	0.91 (0.60 - 1.39)	0.98 (0.70 - 1.38)	1.04 (0.75 - 1.43)	0.90 (0.61 - 1.33)	1.04 (0.75 - 1.42)	Olmesartan	.
1.07 (0.83 - 1.37)	1.23 (0.94 - 1.60)	0.97 (0.77 - 1.23)	1.17 (0.95 - 1.44)	1.08 (0.77 - 1.51)	1.16 (0.92 - 1.46)	1.22 (1.01 - 1.49)	1.06 (0.79 - 1.43)	1.22 (1.01 - 1.49)	1.18 (0.87 - 1.60)	Perindopril
0.87 (0.70 - 1.08)	1.00 (0.79 - 1.26)	0.79 (0.64 - 0.97)	0.95 (0.81 - 1.12)	0.88 (0.64 - 1.20)	0.94 (0.78 - 1.15)	1.00 (0.85 - 1.16)	0.86 (0.66 - 1.13)	0.99 (0.85 - 1.16)	0.96 (0.73 - 1.27)	0.81 (0.72 - 0.92)
0.93 (0.65 - 1.34)	1.07 (0.74 - 1.55)	0.85 (0.59 - 1.21)	1.02 (0.73 - 1.43)	0.94 (0.61 - 1.44)	1.01 (0.71 - 1.44)	1.07 (0.77 - 1.48)	0.93 (0.62 - 1.38)	1.07 (0.77 - 1.48)	1.03 (0.69 - 1.54)	0.87 (0.63 - 1.19)
0.99 (0.78 - 1.25)	1.14 (0.89 - 1.47)	0.90 (0.72 - 1.13)	1.09 (0.90 - 1.32)	1.00 (0.72 - 1.39)	1.08 (0.86 - 1.34)	1.14 (0.94 - 1.37)	0.99 (0.74 - 1.32)	1.13 (0.95 - 1.36)	1.10 (0.81 - 1.47)	0.93 (0.79 - 1.09)
0.97 (0.77 - 1.23)	1.12 (0.87 - 1.43)	0.88 (0.71 - 1.11)	1.07 (0.89 - 1.29)	0.98 (0.71 - 1.36)	1.06 (0.85 - 1.32)	1.12 (0.93 - 1.34)	0.97 (0.73 - 1.29)	1.11 (0.94 - 1.33)	1.08 (0.80 - 1.45)	0.91 (0.78 - 1.06)
0.91 (0.78 - 1.06)	1.05 (0.82 - 1.33)	0.83 (0.64 - 1.07)	1.00 (0.87 - 1.14)	0.92 (0.70 - 1.21)	0.99 (0.77 - 1.27)	1.04 (0.84 - 1.29)	0.91 (0.72 - 1.14)	1.04 (0.89 - 1.23)	1.01 (0.73 - 1.38)	0.85 (0.70 - 1.03)

Shown in each cell are the odds ratio and corresponding 95% confidence interval for pairwise treatment comparisons for MACE. Comparisons in the upper triangle (direct estimates) are presented as row versus column, whereas comparisons in the lower triangle (network estimates) are presented as column versus row.

Table S7. League table of pairwise comparisons for all-cause death

Amlodipine	Atenolol	Candesartan	Captopril	Clortalidone	Enalapril	Irbesartan	Lisinopril	Losartan	Metoprolol	Olmesartan	Perindopril
Amlodipine	.	.	.	.	.	0.97 (0.71 - 1.33)	1.03 (0.89 - 1.20)	.	.	.	.
0.81 (0.60 - 1.08)	Atenolol	.	.	.	.	.	.	1.13 (0.93 - 1.37)	.	.	.
1.05 (0.79 - 1.38)	1.29 (0.93 - 1.81)	Candesartan	.	.	.	.	.	.	.	.	.
1.03 (0.84 - 1.26)	1.27 (1.00 - 1.63)	0.98 (0.75 - 1.30)	Captopril	.	.	.	.	0.90 (0.75 - 1.08)	.	.	.
1.04 (0.84 - 1.29)	1.28 (0.89 - 1.84)	0.99 (0.70 - 1.41)	1.01 (0.75 - 1.35)	Clortalidone	.	.	1.00 (0.86 - 1.16)	.	.	.	.
0.92 (0.70 - 1.20)	1.13 (0.81 - 1.57)	0.87 (0.65 - 1.17)	0.89 (0.68 - 1.16)	0.88 (0.62 - 1.25)	Enalapril	.	.	.	.	.	.
0.98 (0.81 - 1.20)	1.21 (0.91 - 1.62)	0.94 (0.73 - 1.20)	0.95 (0.77 - 1.18)	0.95 (0.71 - 1.27)	1.07 (0.85 - 1.36)	Irbesartan	.	.	.	.	.
1.03 (0.89 - 1.20)	1.28 (0.92 - 1.78)	0.99 (0.72 - 1.36)	1.00 (0.78 - 1.29)	1.00 (0.86 - 1.16)	1.13 (0.83 - 1.54)	1.05 (0.82 - 1.35)	Lisinopril	.	.	.	.
0.91 (0.73 - 1.14)	1.13 (0.93 - 1.37)	0.87 (0.66 - 1.15)	0.89 (0.76 - 1.04)	0.88 (0.65 - 1.20)	1.00 (0.76 - 1.31)	0.93 (0.75 - 1.15)	0.89 (0.68 - 1.16)	Losartan	.	.	.
0.62 (0.34 - 1.12)	0.76 (0.41 - 1.42)	0.59 (0.32 - 1.08)	0.60 (0.33 - 1.08)	0.60 (0.32 - 1.12)	0.68 (0.37 - 1.23)	0.63 (0.35 - 1.12)	0.60 (0.33 - 1.10)	0.68 (0.38 - 1.22)	Metoprolol	.	.
0.67 (0.36 - 1.23)	0.83 (0.44 - 1.56)	0.64 (0.35 - 1.18)	0.65 (0.35 - 1.19)	0.64 (0.34 - 1.23)	0.73 (0.40 - 1.35)	0.68 (0.38 - 1.23)	0.65 (0.35 - 1.21)	0.73 (0.40 - 1.34)	1.08 (0.48 - 2.43)	Olmesartan	.
1.05 (0.83 - 1.32)	1.29 (0.97 - 1.73)	1.00 (0.78 - 1.28)	1.02 (0.81 - 1.27)	1.01 (0.74 - 1.38)	1.14 (0.90 - 1.46)	1.07 (0.88 - 1.29)	1.01 (0.77 - 1.34)	1.15 (0.92 - 1.43)	1.69 (0.95 - 3.02)	1.57 (0.86 - 2.84)	Perindopril
0.98 (0.81 - 1.18)	1.21 (0.93 - 1.57)	0.94 (0.76 - 1.15)	0.95 (0.79 - 1.14)	0.94 (0.71 - 1.25)	1.07 (0.88 - 1.31)	1.00 (0.87 - 1.14)	0.95 (0.75 - 1.21)	1.07 (0.90 - 1.28)	1.58 (0.90 - 2.78)	1.47 (0.82 - 2.61)	0.94 (0.82 - 1.07)
0.99 (0.65 - 1.51)	1.22 (0.77 - 1.93)	0.94 (0.61 - 1.45)	0.96 (0.63 - 1.46)	0.95 (0.59 - 1.53)	1.08 (0.70 - 1.66)	1.00 (0.67 - 1.50)	0.95 (0.61 - 1.50)	1.08 (0.71 - 1.64)	1.59 (0.81 - 3.15)	1.47 (0.74 - 2.95)	0.94 (0.63 - 1.41)
1.04 (0.85 - 1.28)	1.28 (0.97 - 1.70)	0.99 (0.79 - 1.25)	1.01 (0.82 - 1.24)	1.00 (0.74 - 1.35)	1.14 (0.91 - 1.42)	1.06 (0.89 - 1.25)	1.01 (0.78 - 1.30)	1.14 (0.93 - 1.40)	1.68 (0.97 - 2.92)	1.55 (0.86 - 2.80)	0.99 (0.84 - 1.18)
0.99 (0.81 - 1.22)	1.23 (0.93 - 1.62)	0.95 (0.75 - 1.20)	0.96 (0.78 - 1.18)	0.96 (0.71 - 1.29)	1.08 (0.87 - 1.36)	1.01 (0.85 - 1.19)	0.96 (0.74 - 1.25)	1.09 (0.89 - 1.33)	1.60 (0.91 - 2.83)	1.48 (0.82 - 2.67)	0.95 (0.80 - 1.12)
1.02 (0.88 - 1.18)	1.26 (0.97 - 1.64)	0.97 (0.75 - 1.26)	0.99 (0.86 - 1.14)	0.98 (0.76 - 1.28)	1.11 (0.87 - 1.43)	1.04 (0.86 - 1.25)	0.99 (0.80 - 1.22)	1.11 (0.93 - 1.34)	1.65 (0.92 - 2.95)	1.52 (0.84 - 2.77)	0.97 (0.80 - 1.19)

Shown in each cell are the odds ratio and corresponding 95% confidence interval for pairwise treatment comparisons for all-cause death. Comparisons in the upper triangle (direct estimates) are presented as row versus column, whereas comparisons in the lower triangle (network estimates) are presented as column versus row.

Table S8. Meta-regression analyses exploring potencial sources of heterogeneity for MACE, HHF, and all-cause death

Covariate	MACE_Coef_CI	MACE_p_value	HHF_Coef_CI	HHF_p_value	Death_Coe
**Duration of intervention (years)**	-0.0113 (-0.0624 to 0.0398)	0.6643	-0.0023 (-0.0616 to 0.057)	0.9389	-0.0292 (-0.
**Mean Age**	0.0087 (-0.0056 to 0.023)	0.2319	0.0208 (-0.0084 to 0.0499)	0.1625	0.0081 (-0.0
**Female (%)**	0.0023 (0.0002 to 0.0043)	0.0309	0.0032 (-0.0019 to 0.0083)	0.2158	0.0016 (-0.0
**White Ethnicity (%)**	0.0044 (-0.0269 to 0.0357)	0.7827	-0.0091 (-0.0537 to 0.0355)	0,689	-0.0144 (-0.
**Baseline BMI**	0.0084 (-0.0058 to 0.0226)	0.2478	-0.0075 (-0.0362 to 0.0213)	0.6103	0.0152 (0.0
**Baseline eGRF**	0.0003 (-0.0016 to 0.0022)	0.7302	-0.0012 (-0.0043 to 0.0019)	0.4396	0.0009 (-0.0
**Type 2 diabetes (%)**	-0.0001 (-0.002 to 0.0019)	0.9564	2e-04 (-0.0024 to 0.0028)	0.8748	0.0006 (-0.0
**Prior CV Event (%)**	0.0053 (-0.0037 to 0.0143)	0.2491	0.0071 (-0.0069 to 0.0212)	0.3196	0.0024 (-0.0
**Baseline Systolic BP**	-0.0002 (-0.0055 to 0.0051)	0.938	0.0045 (-0.0017 to 0.0107)	0.1568	0.003 (-0.00

Table S9. References of randomized controlled trials included in the network meta-analysis

RCT	First author	publishing year	Journal	Reference
AASK	Wright, Jr	2002	JAMA	Wright, Jr, et al. JAMA 2002;288:2421-2431
ALLHAT	Wright, Jr	2002	JAMA	Wright, Jr, et al. JAMA 2002;288:2981-2997
AMADEO	Bakris	2008	Kidney International	Bakris, et al. Kidney International 2008;74:364-369
CONSENSUS II	Swedberg	1992	N Engl J Med	Swedberg, et al. N Engl J Med 1992;327:678-684
DIABHYCAR	Marre	2004	BMJ	Marre M, et al. BMJ 2004;328:686
EUROPA	Fox	2003	Lancet	Fox KM, et al. Lancet 2003;362:782-788
GISSIAF	Disertori	2009	N Engl J Med	Disertori M, et al. N Engl J Med 2009;360:1606-1617
HOPE	Yusuf	2000	N Engl J Med	Yusuf S, et al. N Engl J Med 2000;342:145-153
IDNT	Lewis	2001	N Engl J Med	Lewis EJ, et al. N Engl J Med 2001;345:851-860
IMAGINE	Rouleau	2008	Circulation	Rouleau JL, et al. Circulation 2008;117:24-31
Kondo et al., 2003	Kondo	2003	Am Heart J	Kondo J, et al. Am Heart J 2003;146:e20
LIFE	Dahlof	2002	Lancet	Dahlof B, et al. Lancet 2002;359:995-1003
NAVIGATOR	McMurray	2010	N Engl J Med	McMurray JJ, et al. N Engl J Med 2010;362:1477-1490
OLIVUS-Ex	Hirohata	2012	Atherosclerosis	Hirohata A, et al. Atherosclerosis 2012;220:134-138
ONTARGET	Yusuf	2008	N Engl J Med	Yusuf A, et al. N Engl J Med 2008;358:1547-1459
OPTIMAAL	Dickstein	2002	Lancet	Dickstein K, et al. Lancet 2002;360:752-760
PART-2	MacMahon	2000	JACC	MacMahon S, et al. JACC 2000;36:438-443
PREAMI	Ferrari	2006	Arch Intern Med	Ferrari R, et al. Arch Intern Med 2006;166:659-666
PROGRESS	MacMahon	2001	Lancet	MacMahon S, et al. Lancet 2001;358:1033-1041
PRoFESS	Yusuf	2008	N Endl J Med	Yusuf S, et al. N Engl J Med 2008;359:1225-1237
QUIET	Pitt	2001	Am J Cardiol	Pitt B, et al. Am J Cardiol 2001;87:1058-1063
ROADMAP	Haller	2011	N Engl J Med	Haller H, et al. N Engl J Med 2011;364:907-917
SCAT	Teo	2000	Circulation	Teo KK, et al. Circulation 2000;102:1748-1754
SCOPE	Lithell	2003	Journal of Hypertension	Lithell H, et al. Journal of Hypertension 2003:875-886
TRANSCEND	Yusuf	2008	Lancet	Yusuf S, et al. Lancet 2008;372:1174-1183
RCT	First author	publishing year	Journal	Reference
VALUE	Julius	2004	Lancet	Julius S, et al. Lancet 2004;363:2022-2031

VIVALD	Galle	2008	Nephrol Dial Transplant	Galle J, et al. Nephrol Dial Transplant 2008;23:3174-3183

Table S7. PRISMA NMA Checklist of Items to Include When Reporting A Systematic Review Involving a Network Meta- analysis

Section/Topic	Item #	Checklist Item	Location where item is reported
**TITLE**			
Title	1	Identify the report as a systematic review *incorporating a network meta-analysis (or related form of meta-analysis).*	Page 1
**ABSTRACT**			Pages 2-3
Structured summary	2	Provide a structured summary including, as applicable:<br>**Background:** main objectives<br>**Methods:** data sources; study eligibility criteria, participants, and interventions; study appraisal; and *synthesis methods, such as network meta-analysis.*<br>**Results:** number of studies and participants identified; summary estimates with corresponding confidence/credible intervals; *treatment rankings may also be discussed. Authors may choose to summarize pairwise comparisons against a chosen treatment included in their analyses for brevity.*<br>**Discussion/Conclusions:** limitations; conclusions and implications of findings.<br>**Other:** primary source of funding; systematic review registration number with registry name.	
**INTRODUCTION**			
Rationale	3	Describe the rationale for the review in the context of what is already known*, including mention of why a network meta-analysis has been conducted.*	Page 3
Objectives	4	Provide an explicit statement of questions being addressed, with reference to participants, interventions, comparisons, outcomes, and study design (PICOS).	Pages 3-4
**METHODS**			
Protocol and registration	5	Indicate whether a review protocol exists and if and where it can be accessed (e.g., Web address); and, if available, provide registration information, including registration number.	Page 6
Eligibility criteria	6	Specify study characteristics (e.g., PICOS, length of follow-up) and report characteristics (e.g., years considered, language, publication status) used as criteria for eligibility, giving rationale. *Clearly describe eligible treatments included in the treatment network, and note whether any have been clustered or merged into the same node (with justification).*	Page 6
Information sources	7	Describe all information sources (e.g., databases with dates of coverage, contact with study authors to identify additional studies) in the search and date last searched.	Page 6
Search	8	Present full electronic search strategy for at least one database, including any limits used, such that it could be repeated.	Page 6,7
Study selection	9	State the process for selecting studies (i.e., screening, eligibility, included in systematic review, and, if applicable, included in the meta-analysis).	Page 6
Data collection process	10	Describe method of data extraction from reports (e.g., piloted forms, independently, in duplicate) and any processes for obtaining and confirming data from investigators.	Page 7
Data items	11	List and define all variables for which data were sought (e.g., PICOS, funding sources) and any assumptions and simplifications made.	Page 6
**Geometry of the network**	**S1**	Describe methods used to explore the geometry of the treatment network under study and potential biases related to it. This should include how the evidence base has been graphically summarized for presentation, and what characteristics were compiled and used to describe the evidence base to readers.	Page 6-7
Risk of bias within individual studies	12	Describe methods used for assessing risk of bias of individual studies (including specification of whether this was done at the study or outcome level), and how this information is to be used in any data	Page 7

		synthesis.	
Summary measures	13	State the principal summary measures (e.g., risk ratio, difference in means). *Also describe the use of additional summary measures assessed, such as treatment rankings and surface under the cumulative ranking curve (SUCRA) values, as well as modified approaches used to present summary findings from meta-analyses.*	Page 8
Planned methods of analysis	14	Describe the methods of handling data and combining results of studies for each network meta-analysis. This should include, but not be limited to:<br>• *Handling of multi-arm trials;*<br>• *Selection of variance structure;*<br>• *Selection of prior distributions in Bayesian analyses; and*<br>• *Assessment of model fit.*	Page 8
**Assessment of Inconsistency**	**S2**	Describe the statistical methods used to evaluate the agreement of direct and indirect evidence in the treatment network(s) studied. Describe efforts taken to address its presence when found.	Page 8
Risk of bias across studies	15	Specify any assessment of risk of bias that may affect the cumulative evidence (e.g., publication bias, selective reporting within studies).	Page 10
Additional analyses	16	Describe methods of additional analyses if done, indicating which were pre-specified. This may include, but not be limited to, the following:<br>• Sensitivity or subgroup analyses;<br>• Meta-regression analyses;<br>• *Alternative formulations of the treatment network; and*<br>• *Use of alternative prior distributions for Bayesian analyses (if applicable).*	Page 9

RESULTS†

Study selection	17	Give numbers of studies screened, assessed for eligibility, and included in the review, with reasons for exclusions at each stage, ideally with a flow diagram.	Page 10
**Presentation of network structure**	**S3**	Provide a network graph of the included studies to enable visualization of the geometry of the treatment network.	Figure S2
**Summary of network geometry**	**S4**	Provide a brief overview of characteristics of the treatment network. This may include commentary on the abundance of trials and randomized patients for the different interventions and pairwise comparisons in the network, gaps of evidence in the treatment network, and potential biases reflected by the network structure.	Page 10
Study characteristics	18	For each study, present characteristics for which data were extracted (e.g., study size, PICOS, follow-up period) and provide the citations.	Table S1 and Table S6
Risk of bias within studies	19	Present data on risk of bias of each study and, if available, any outcome level assessment.	Table S2
Results of individual studies	20	For all outcomes considered (benefits or harms), present, for each study: 1) simple summary data for each intervention group, and 2) effect estimates and confidence intervals. *Modified approaches may be needed to deal with information from larger networks.*	Forest plots (Figures 1a, 1b and 1c)
Synthesis of results	21	Present results of each meta-analysis done, including confidence/credible intervals. *In larger networks, authors may focus on comparisons versus a particular comparator (e.g. placebo or standard care), with full findings presented in an appendix. League tables and forest plots may be considered to summarize pairwise comparisons.* If additional summary measures were explored (such as treatment rankings), these should also be presented.	Pages 10-12
**Exploration for inconsistency**	**S5**	Describe results from investigations of inconsistency. This may include such information as measures of model fit to compare consistency and inconsistency models, *P* values from statistical tests, or summary of inconsistency estimates from different parts of the treatment network.	Page 8, Page 10
Risk of bias across studies	22	Present results of any assessment of risk of bias across studies for the evidence base being studied.	Table S3, Figure S3

Results of additional analyses	23	Give results of additional analyses, if done (e.g., sensitivity or subgroup analyses, meta-regression analyses*, alternative network geometries studied, alternative choice of prior distributions for Bayesian analyses,* and so forth).	Pages 12-14
**DISCUSSION**			
Summary of evidence	24	Summarize the main findings, including the strength of evidence for each main outcome; consider their relevance to key groups (e.g., healthcare providers, users, and policy-makers).	Pages 15-16
Limitations	25	Discuss limitations at study and outcome level (e.g., risk of bias), and at review level (e.g., incomplete retrieval of identified research, reporting bias). *Comment on the validity of the assumptions, such as transitivity and consistency. Comment on any concerns regarding network geometry (e.g., avoidance of certain comparisons).*	Pages 16-17
Conclusions	26	Provide a general interpretation of the results in the context of other evidence, and implications for future research.	Pages 17-18
**FUNDING**			Page 19
Funding	27	Describe sources of funding for the systematic review and other support (e.g., supply of data); role of funders for the systematic review. This should also include information regarding whether funding has been received from manufacturers of treatments in the network and/or whether some of the authors are content experts with professional conflicts of interest that could affect use of treatments in the network.	

PICOS = population, intervention, comparators, outcomes, study design.
