# Supplementary material for: Efeitos dos Diferentes Bloqueadores dos Receptores da Angiotensina II Sobre Eventos Cardiovasculares Adversos Maiores: Revisão Sistemática e Metanálise em Rede
Source: Arq Bras Cardiol. 2026 Oct 21;123(9):e20260274. [Article in Portuguese] doi: 10.36660/abc.20260274 (PMC13754392; doi:10.36660/abc.20260274)
Supplement: Translation

# Translation: Individual Effects of Angiotensin II Receptor Blockers on Major Adverse Cardiovascular Events: A Systematic Review and Network Meta-Analysis

**DOI:** 10.36660/abc.20260274i

**Authors:** Eurico Joaquim Lutucuta Ernesto, Ana Claudia Cavalcante Nogueira, Luis Gustavo Sedenho-Prado, Louise Buonalumi Tácito Yugar, Marília Paiva Martins, Daniel Campos Jesus, Jairo Lins Borges, Wilson Nadruz, Luiz Sérgio F Carvalho, Andrei C. Sposito

**Affiliations:** 1 Brazil Universidade Estadual de Campinas (UNICAMP), Campinas, SP – Brazil; 2 Brazil Universidade de Brasília, Brasília, DF – Brazil; 3 Brazil Hospital de Base do Distrito Federal, Brasília, DF – Brazil; 4 Brazil Instituto Aramari Apo, Brasília, DF – Brazil; 5 Brazil Universidade Federal de São Paulo (UNIFESP), São Paulo, SP – Brazil; 6 Brazil Universidade Católica de Brasília, Taguatinga, DF – Brazil

**Keywords:** Angiotensin II Type 1 Receptor Blockers, Cardiovascular Diseases, Network Meta-Analysis

## Abstract

**Background:**

Angiotensin II receptor blockers (ARBs) antagonize the AT1 receptor and are effective antihypertensives. Modest differences in blood pressure-lowering efficacy have been reported, reflecting variations in pharmacokinetics, receptor binding, duration of action, and potential pleiotropic effects. However, data on their comparative effects on cardiovascular outcomes in patients are scarce.

**Objective:**

To assess the effects of ARBs on MACE, hospitalization for heart failure (HHF), and all-cause mortality in randomized trials not primarily designed to evaluate heart failure (HF).

**Methods:**

MEDLINE/PubMed, Cochrane Central Register of Controlled Trials, and ClinicalTrials.gov databases were searched until May 2025. Published RCTs reporting major adverse cardiovascular events (MACE), HHF or all-cause death, involving ARBs, with at least 100 individuals per arm and ≥12 months of follow-up, were included. Studies focusing primarily on patients with existing HF were excluded. Incidence rates of HHF, MACE, and all-cause death. A random-effects network meta-analysis including 27 RCTs and 702,584 patient-years was performed.

**Results:**

Candesartan reduced MACE versus placebo (OR 0.79; 95% CI, 0.63–0.97; SUCRA=0.85), ranking highest among ARBs, while telmisartan showed a smaller but significant reduction (OR 0.90; 95% CI, 0.80–0.99; SUCRA=0.61). The analysis relied on study-level data; included moderately sparse treatment networks with predominantly indirect comparisons between ARBs.

**Conclusion:**

This network meta-analysis in patients without baseline HF indicates that candesartan and telmisartan reduce MACE, suggesting clinically meaningful differences among ARBs. Given the predominantly indirect nature of several comparisons, these findings should be interpreted cautiously.

## Introduction

Angiotensin II receptor blockers (ARBs) are a pharmacologic class characterized by selective antagonism of the angiotensin II type 1 receptor (AT_1_R). Although all ARBs effectively lower blood pressure (BP), head-to-head trials and meta-analyses suggest modest differences in antihypertensive efficacy and 24-hour BP control.^1^ Clinically relevant differences have also been observed in their pharmacokinetic properties, receptor-binding characteristics, duration of action, and potential pleiotropic effects.^1-3^ For example, candesartan exhibits insurmountable antagonism and a slow dissociation rate, resulting in receptor blockade that persists beyond its plasma half-life. In contrast, losartan acts as a competitive antagonist, whereas its active metabolite (i.e., EXP 3174), as well as irbesartan and valsartan, exhibits intermediate antagonistic profiles. Structural analyses and molecular dynamics simulations have shown that, despite sharing a common binding site on the AT_1_R, ARBs exhibit distinct spatiotemporal interactions and binding patterns that may explain differences in their pharmacodynamic properties and clinical efficacy.^3^ These distinctions are supported by in vitro vascular assays and radioligand-binding studies and may influence clinical outcomes, including the duration of therapeutic effects and BP control.^2,4-6^

Excessive inflammation also contributes to adverse outcomes in cardiovascular disease. ARBs and angiotensin-converting enzyme (ACE) inhibitors have demonstrated protective effects on the coronary endothelium and pulmonary epithelium, particularly among patients with acute inflammatory conditions or viral infections. These pleiotropic effects may contribute to differences in clinical efficacy beyond BP control. This systematic review and network meta-analysis (NMA) aimed to compare the effects of individual ARBs on the risks of hospitalization for heart failure (HF), major adverse cardiovascular events (MACE), and all-cause mortality.

## Methods

This systematic review was conducted in accordance with the Preferred Reporting Items for Systematic Reviews and Meta-Analyses guidelines. The protocol was registered in PROSPERO under registry code CRD42024596628. Because this meta-analysis used only data extracted from previously published studies, institutional review board approval and informed consent were not required.

### Search strategy and study eligibility

Details of the data sources and search strategy are provided in the Supplementary Material. Eligibility criteria were established using the population/intervention/comparator/outcome framework: the population comprised patients with hypertension or high cardiovascular risk without HF at baseline; the interventions were individual ARBs; the comparators were placebo or ACE inhibitors; and the outcomes were MACE, hospitalization for HF, and all-cause mortality.

We searched MEDLINE via PubMed, ClinicalTrials.gov, the Cochrane Central Register of Controlled Trials, Embase, the European Union Clinical Trials Register, and the World Health Organization International Clinical Trials Registry Platform from database inception through May 2025. Inclusion was restricted to full-text randomized clinical trials (RCTs). The initial search strategy combined terms related to hypertension and antihypertensive therapy. For the present analysis, eligibility was narrowed to trials evaluating ARBs.

The original protocol focused on comparisons among ARBs. However, restricting the NMA exclusively to trials of ARBs resulted in two disconnected subnetworks, limiting indirect comparisons among several agents. After inspection of the network geometry, trials with ACE inhibitor comparators were incorporated to create a connected evidence network and enable indirect comparisons among ARBs while preserving the primary focus on ARB therapy. This approach relied on the assumptions of transitivity and similarity between trials of ARBs and ACE inhibitors with respect to baseline population characteristics, cardiovascular risk, and therapeutic targets. The inclusion of ACE inhibitor comparators was also considered biologically plausible because both treatment classes modulate the renin-angiotensin-aldosterone system.

Studies were eligible if they i) used a randomized, double-blind, controlled design; ii) evaluated an ARB or ACE inhibitor; iii) reported MACE, hospitalization for HF, or all-cause mortality; iv) had a follow-up duration of at least 12 months; and v) enrolled at least 100 participants in any randomized group. Trials primarily designed to evaluate HF were excluded to reduce clinical heterogeneity. Outcome definitions are provided in Table S4.

### Data extraction and quality assessment

Details of the data extraction and quality assessment procedures are provided in the Supplementary Material. Data were independently extracted by five reviewers (EJLE, LGSP, LBTY, MPM, and DCJ) and subsequently reviewed by ACN. Discrepancies were resolved through discussion. Extracted data included study characteristics, participant baseline characteristics, and clinical outcomes.

Study quality and risk of bias were assessed using the Cochrane Risk of Bias 2 (RoB 2) tool. The certainty of evidence for each outcome was assessed using the Grading of Recommendations Assessment, Development and Evaluation (GRADE) approach adapted for NMA. Evidence was evaluated across 5 domains: risk of bias, inconsistency, indirectness, imprecision, and publication bias. Certainty of evidence was classified as high, moderate, low, or very low.

### Data synthesis and statistical analysis

The primary outcome was MACE, defined as a composite of myocardial infarction, stroke, and cardiovascular death. Secondary outcomes were hospitalization for HF and all-cause mortality. An NMA was conducted to estimate direct and indirect comparisons among treatments using the “discomb” package in R.

Categorical variables are reported as numbers and percentages, whereas continuous variables are reported as mean ± SD or median (IQR) as appropriate. Baseline characteristics were calculated using weighted estimates. Treatment effects were expressed as odds ratios (ORs) for the random-effects NMA and risk ratios for random-effects pairwise meta-analyses.

The surface under the cumulative ranking curve (SUCRA) was calculated for each treatment and outcome. Relative treatment rankings were summarized using SUCRA values, which reflected the probability that each intervention was among the most effective treatments. Rankograms were generated to display the distribution of ranking probabilities across treatments for each outcome. Pairwise treatment effects were also summarized in league tables presenting ORs and corresponding 95%CIs for all available comparisons within the NMA.

Statistical heterogeneity among trials was assessed using the Higgins I^2^ statistic with 95%CIs. The I^2^ statistic was derived from Cochran Q as 100 × (Q − df)/Q and represented the proportion of total variation attributable to between-trial heterogeneity. Because of the moderately sparse network geometry and the limited number of closed loops for several treatment comparisons, inconsistency was assessed qualitatively by inspecting the network structure, comparing direct and indirect estimates when feasible, and evaluating the stability of the results across sensitivity analyses. Formal statistical tests of inconsistency were not performed.

Potential sources of heterogeneity among RCTs were investigated using meta-regression analyses. Prespecified covariates included sample size, intervention duration, age, sex, race and ethnicity, body mass index (BMI), chronic kidney disease (CKD) diagnosis and stage, previous cardiovascular events, and baseline systolic BP (SBP). Publication bias was assessed using funnel plots and the Egger test.

The robustness of the findings was evaluated by repeating the NMA under alternative assumptions. First, RCTs comparing ACE inhibitors with placebo were excluded. Second, a leave-random-25%-out sensitivity analysis was conducted for each outcome. In this analysis, six trials reporting all-cause mortality, six reporting MACE, and five reporting hospitalization for HF were randomly excluded, after which the pooled ORs and corresponding 95%CIs were recalculated. Meta-regression analyses were also conducted to investigate whether differences in achieved BP between the active-treatment and control groups were associated with the incidence of clinical outcomes for each drug.

All statistical tests were two-sided, and p < 0.05 was considered statistically significant. Data extraction and quality assessments underwent additional review, and remaining discrepancies were resolved through discussion with senior researchers. Analyses were performed using R, version 4.0.1 (R Foundation for Statistical Computing), with the “discomb” (version 1.0.0), “metaviz” (version 0.3.1), and “metafor” (version 2.4.0) packages.

## Results

Database searches identified a total of 3,642 records. After 347 duplicates were removed, 3,295 records remained for screening. Of these, 2,242 were excluded after title and abstract screening, leaving 1,053 reports for full-text assessment. After full-text review, 27 RCTs were included (Figure S1). The number of trials contributing to each network varied according to outcome reporting: 27 contributed to the MACE analysis, 19 to the analysis of hospitalization for HF, and 27 to the all-cause mortality analysis.

The baseline characteristics of participants in the included RCTs are presented in Table S1. Definitions and components of MACE across the included trials, including whether MACE was designated as a primary or secondary outcome in each study, are summarized in Table S4. Most studies were assessed as having a low risk of bias using the RoB 2 tool (Table S2), and most outcome-specific comparisons were rated as having high-certainty evidence using the GRADE approach (Table S5). The Egger tests indicated no significant publication bias for hospitalization for HF (p = 0.16), MACE (p = 0.22), or all-cause mortality (p = 0.16) (Table S3 and Figures S3A-S3C).

The evidence networks for MACE, hospitalization for HF, and all-cause mortality were predominantly centered on placebo-controlled comparisons, with relatively few direct head-to-head comparisons among ARBs (Figure S2). Consequently, most comparative estimates were derived indirectly through placebo or, to a lesser extent, ACE inhibitor comparators. Although placebo, losartan, ramipril, telmisartan, and valsartan were the most highly connected treatment nodes, evidence for several ARBs was derived from a limited number of studies. This resulted in moderately sparse network geometry and greater reliance on indirect evidence for some pairwise comparisons. Qualitative assessment of the network geometry and coherence of effect estimates revealed no major inconsistency within the analyzed networks. The complete list of included RCTs and their corresponding references is provided in Table S1.

The meta-analysis included 174,873 participants, with a mean follow-up of 3.1 ± 1.4 years for the primary outcome, totaling 702,584 patient-years. The mean participant age was 65 ± 4 years; 33% of participants were female, and 67% were White. Participants had been diagnosed with hypertension for a mean of 9 ± 4 years. In addition, 35% had type 2 diabetes mellitus, and 46% had dyslipidemia. Overall, 48% of participants were enrolled in secondary prevention trials, primarily because of coronary artery disease or previous stroke, whereas 52% were enrolled in primary prevention trials.

At baseline, the mean BMI across trials was 28.5 ± 4.1 kg/m^2^, mean SBP was 141 ± 14 mmHg, mean diastolic BP was 82 ± 7 mmHg, mean left ventricular ejection fraction (LVEF) was 56% ± 9%, and mean estimated glomerular filtration rate was 73 ± 11 mL/min/1.73 m^2^.

In addition to ARBs, comparator data were available from RCTs evaluating amlodipine, atenolol, chlorthalidone, and ACE inhibitors. Because the search strategy was not designed to comprehensively identify trials of these agents, the available evidence was insufficient to assess their individual effects. Analyses that included these comparators were therefore considered exploratory. Corresponding forest plots are presented in Figure S5 for transparency.

### Primary outcome: major adverse cardiovascular events

The analysis included 27 trials and showed moderate heterogeneity (I^2^ = 41%). Compared with placebo, candesartan was associated with a significant reduction in MACE (OR, 0.79; 95%CI, 0.63-0.97; p = 0.03; SUCRA = 0.85). Telmisartan was also associated with a smaller but significant reduction in MACE (OR, 0.90; 95%CI, 0.80-0.99; p = 0.02; SUCRA = 0.61). No other agent was associated with a significant reduction in MACE (Figure 1A).

**Figure 1:** – Forest plots of MACE, hospitalization for HF, and all-cause mortality. MACE: major adverse cardiovascular events; HF: heart failure; OR: odds ratio.

### Secondary outcomes

The NMA of hospitalization for HF included 19 trials and showed no statistical heterogeneity (I^2^ = 0%). Qualitative assessment revealed no major inconsistency between the direct and indirect evidence. Although irbesartan had only a moderate probability of benefit based on its treatment ranking (SUCRA = 0.46), it was the only agent associated with a significant reduction in hospitalization for HF (OR, 0.87; 95%CI, 0.78-0.98; p = 0.02). This finding should be interpreted cautiously because it was driven predominantly by a single trial and was supported by moderate-certainty evidence according to the GRADE assessment.

The analysis of all-cause mortality included 27 trials and showed low to moderate heterogeneity (I^2^ = 38%). No treatment was associated with a significant difference in all-cause mortality compared with placebo (Figure 1C).

Rankograms for MACE and all-cause mortality are presented in Figure S9, and complete pairwise comparisons are provided in Tables S6 and S7.

### Sensitivity analysis and meta-regression analyses

Because of the moderate heterogeneity observed for MACE and the diversity of the populations, comparators, and study designs across the included trials, multiple sensitivity analyses were conducted to assess the robustness of the primary findings.

As shown in Figure S4, the leave-random-25%-out sensitivity analysis produced results consistent with those of the primary analysis across all outcomes. Similarly, restricting the analysis to RCTs at low risk of bias according to the RoB 2 tool did not materially alter the results.

In another sensitivity analysis, trials comparing ACE inhibitors with placebo were excluded (Figure S6). After these trials were excluded, candesartan remained the only agent significantly associated with a lower risk of MACE compared with placebo (OR, 0.79; 95%CI, 0.66-0.96). For hospitalization for HF and all-cause mortality, excluding these trials reduced statistical power, and no agent was significantly associated with either outcome.

Using a fixed-effect model instead of a random-effects model produced estimates consistent with those of the primary analysis, including for hospitalization for HF, for which no statistical heterogeneity was observed (I^2^ = 0%).

Meta-regression analyses were conducted to investigate whether the magnitude of SBP reduction achieved in the active-treatment groups relative to the control groups influenced clinical outcomes. Visual inspection of the meta-regression plots for all-cause mortality and MACE suggested that the treatment effects of most individual ARBs, including telmisartan, valsartan, and irbesartan, remained relatively stable across the observed range of between-group differences in SBP (Figure S7). Although candesartan and olmesartan showed steeper slopes in these exploratory analyses, the wide CIs indicated substantial uncertainty, and the observed associations were generally not statistically significant.

Furthermore, as shown in Figure S8, forest plots stratified by the magnitude of SBP change (e.g., reduction > - 1.3 mmHg, -1.3 to -2.9 mmHg, and < -2.9 mmHg)demonstrated that the risks of all-cause mortality and MACE remained largely consistent regardless of the achieved difference in BP. The overall pooled effect for MACE reached statistical significance only in the subgroup with the greatest reduction in SBP (hazard ratio, 0.92; 95%CI, 0.87-0.97), suggesting that BP reduction may have partially contributed to the observed treatment effects.

Furthermore, extensive meta-regression analyses showed that other prespecified factors (e.g., sample size, intervention duration, age, sex, race and ethnicity, BMI, CKD diagnosis and stage, and previous cardiovascular events) did not significantly influence the results for the primary or secondary outcomes (Table S8). These findings support the robustness of the benefits observed with candesartan and telmisartan, suggesting that these benefits may be attributable to intrinsic pharmacologic properties rather than to baseline participant characteristics or the degree of BP control alone.

## Discussion

This NMA provides a quantitative assessment of the associations between individual ARBs and MACE in RCTs not specifically designed to evaluate HF. Candesartan and telmisartan were associated with significant reductions in MACE, whereas irbesartan was associated with a reduction in hospitalization for HF in exploratory analyses. However, the finding for irbesartan should be interpreted cautiously because it was driven predominantly by a single trial involving patients with diabetes and marked proteinuria, limiting its generalizability. In addition, the certainty of evidence for this outcome was downgraded to moderate in the GRADE assessment. No agent demonstrated a significant mortality benefit. These findings raise the hypothesis that pharmacodynamic or pharmacokinetic differences among ARBs may be associated with differences in clinical outcomes.

Prior meta-analyses have primarily focused on the BP-lowering efficacy of individual ARBs or evaluated ARBs as a class in relation to cardiovascular and renal outcomes.^7-10^ Although some studies quantified differences in BP reduction, such as the less effective ambulatory BP control observed with losartan,^7^ none directly compared cardiovascular outcomes among individual ARBs. To our knowledge, this is the first NMA to assess differences in MACE among individual ARBs.

As previously noted, distinct spatiotemporal interactions and binding patterns have been described among ARBs.^3^ These differences may influence both the duration of therapeutic effects and BP control.^2,4-6^ Although all ARBs selectively block the AT_1_R, their affinity for this receptor varies substantially. For example, candesartan exhibits more than 10,000-fold selectivity for the AT_1_R over the angiotensin II type 2 receptor.^6^ It acts as an insurmountable antagonist, producing sustained, noncompetitive blockade of angiotensin II-induced vasoconstriction.^5,6^ In contrast, losartan acts as a competitive antagonist, producing a parallel rightward shift in the angiotensin II dose-response curve without reducing the maximal response.^6^ Irbesartan and valsartan exhibit properties intermediate between competitive and insurmountable antagonism.^5,6^ Whether these pharmacologic distinctions or other as-yet unidentified mechanisms underlie the differences observed in this study remains uncertain. Nevertheless, the present findings suggest that pharmacologic differences among ARBs may translate into clinically meaningful differences in outcomes.

This study has several strengths, including the use of an NMA framework, which enabled broad comparisons across treatments, and its specific focus on patients without HF at baseline. Nonetheless, several important limitations should be acknowledged. First, the analysis was based on study-level rather than individual participant-level data, precluding detailed subgroup analyses. Second, although statistical heterogeneity was moderate for MACE, substantial clinical and methodological variability remained across the included trials Despite the consistency of the results across multiple sensitivity analyses, residual heterogeneity cannot be excluded. Third, the treatment networks were based predominantly on placebo-controlled comparisons, with relatively few direct head-to-head comparisons among ARBs, resulting in moderately sparse network geometry. In addition, trials with ACE inhibitor comparators were incorporated to preserve network connectivity. This approach may have increased reliance on indirect evidence and warrants cautious interpretation of some comparisons. Fourth, variations in study design, follow-up duration, and outcome definitions across RCTs may have influenced the findings. Fifth, although trials specifically designed to evaluate HF were excluded, baseline LVEF and asymptomatic left ventricular dysfunction were not uniformly reported. Consequently, residual differences in ventricular function across studies cannot be excluded, and potential effect modification according to baseline LVEF could not be systematically assessed. Sixth, although SUCRA rankings provide a probability-based hierarchy of treatment effectiveness, they should be interpreted cautiously. High SUCRA values do not necessarily indicate clinically meaningful superiority, particularly when effect sizes are modest and CIs overlap. Seventh, exploratory meta-regression analyses suggested that the magnitude of BP reduction did not fully explain the observed differences in outcomes among ARBs. However, BP reduction may have acted as a partial mediator, and its role cannot be excluded because of the inherent limitations of study-level analyses. Finally, as with all meta-analyses, null findings may reflect a lack of adequately designed RCTs rather than a true absence of treatment effect.

## Conclusion

This NMA suggests clinically meaningful differences among ARBs in patients without HF at baseline, with candesartan and, to a lesser extent, telmisartan associated with reductions in MACE. Because of the predominantly indirect nature of several comparisons and the moderately sparse network geometry, these findings should be interpreted cautiously and considered hypothesis-generating rather than definitive evidence supporting preferential selection of a specific ARB. Nevertheless, the observed differences in outcomes among ARBs are consistent with the pharmacologic diversity of this drug class recognized in current guidelines.

## Supplementary Material

*Supplemental Materials For additional information, please click here.http://abccardiol.org/supplementary-material/2026/12309/2026-0274-supplement-data.pdf
